# Diverse toxin repertoire but limited metabolic capacities inferred from the draft genome assemblies of three Spiroplasma (Citri clade) strains associated with Drosophila

**DOI:** 10.1099/mgen.0.001408

**Published:** 2025-06-05

**Authors:** Paulino Ramirez, Humberto Martinez Montoya, Rodolfo Aramayo, Mariana Mateos

**Affiliations:** 1 Dept. Wildlife and Fisheries Sciences, USA, Texas A&M University, College Station, Texas; 2 Dept. Ecology and Conservation Biology, Texas A&M University, College Station, Texas, USA; 3 Center for Alzheimer’s Disease Research, Carney Institute for Brain Science, Brown University, Providence, Rhode Island, USA; 4 University of Texas Health Center at San Antonio, San Antonio, Texas, USA; 5 Unidad Academica Multidisciplinaria Reynosa Aztlán - Universidad Autónoma de Tamaulipas, Reynosa, Tamaulipas, Mexico; 6 Dept. Biology, Texas A&M University, College Station, Texas, USA

**Keywords:** *Mycoplasma*, phylogeny, symbiosis, vertical transmission

## Abstract

*Spiroplasma* (class *Mollicutes*) is a diverse wall-less bacterial genus whose members are strictly dependent on eukaryotic hosts (mostly arthropods and plants), with which they engage in pathogenic to mutualistic interactions. *Spiroplasma* are generally fastidious to culture *in vitro*, especially those that are vertically transmitted by their hosts, which include flies in the genus *Drosophila*. *Drosophila* has been invaded by at least three independent clades of *Spiroplasma*: Poulsonii (the best studied, contains reproductive manipulators and defensive mutualists associated with two major clades of *Drosophila* and has amongst the highest substitution rates within bacteria), Citri (restricted to the *repleta* group of *Drosophila*) and Ixodetis. We report the first genome drafts of *Drosophila*-associated Citri clade *Spiroplasma*: strain *s*Moj from *Drosophila mojavensis*, strain *s*Ald-Tx from *Drosophila aldrichi* from Texas (newly discovered; also associated with *Drosophila mulleri*) and strain *s*Hy2 from *Drosophila hydei* (the only *Drosophila* species known to naturally also harbour a Poulsonii clade strain, thereby providing an arena for horizontal gene transfer). Compared to their Poulsonii clade counterparts, we infer that the three Citri clade strains have the following: (1) equal or worse DNA repair abilities; (b) more limited metabolic capacities, which may underlie their comparatively lower titres and transmission efficiency; and (c) similar content of toxin domains, including at least one ribosome-inactivating protein, which is implicated in the Poulsonii-conferred defence against natural enemies. As a byproduct of our phylogenomic analyses and exhaustive search for certain toxin domains in public databases, we document the toxin repertoire in close relatives of *Drosophila*-associated *Spiroplasma*, and in a very divergent newly discovered lineage (i.e. ‘clade X’). Phylogenies of toxin-encoding genes or domains imply substantial exchanges between closely and distantly related strains. Surprisingly, despite encoding several toxin genes and achieving relatively high prevalences in certain natural populations (*s*Ald-Tx in this study; *s*Moj in prior work), fitness assays of *s*Moj (this study) and *s*Ald-Tx (prior work) in the context of wasp parasitism fail to detect a beneficial effect to their hosts. Thus, how Citri clade strains persist in their *Drosophila* host populations remains elusive.

## Data Summary

All novel sequencing data are available through National Center for Biotechnology Information (NCBI) repositories. Illumina raw reads, assemblies and NCBI annotations are available under BioProject nos. PRJNA506493 for *s*Hy2, PRJNA506491 for *s*Ald-Tx and PRJNA355307 for *s*Moj. Oxford Nanopore (MinION) reads for *s*Hy2 are under SRA Accession Number SRR12348752.

Supporting Material is available under the DOI 10.6084 /m9.figshare.c.7437997 or as accompanying supporting documents in the corresponding preprint server or scientific journal.

**Impact Statement**

Symbiotic associations between arthropods and inherited microbes are pervasive, taxonomically and mechanistically diverse and strongly influential. Research into the mechanisms and processes governing such heritable interactions is hindered by our inability to culture most inherited symbionts outside of their hosts. We studied three heritable strains of *Spiroplasma* (Citri clade) that naturally associate with *Drosophila* flies and that reach relatively high prevalence in certain host populations but appear to lack traits that would enable them to persist in host populations, such as high vertical transmission efficiency, reproductive manipulation or fitness benefits. We compared their genomes to those of a separate *Spiroplasma* clade (Poulsonii) that associates with *Drosophila*, which does exhibit some of the traits that contribute to persistence, including protection against natural enemies of their hosts, and also has amongst the highest DNA substitution rates recorded for bacteria. Compared to Poulsonii, the three Citri clade strains have smaller genomes and fewer genes, leading us to predict they have similarly high DNA substitution rates, but more limited metabolic capacities, which may explain the comparatively lower densities that they achieve within individual hosts, and their frequent loss in lab colonies of their hosts. However, the toxin repertoire of the Citri clade was comparatively diverse, and the result of horizontal gene exchange amongst close and distant strains, and within-genome shuffling. We hypothesize that Citri clade strains persist via unknown fitness benefits conferred to their hosts (possibly mediated by toxins) or by substantial horizontal transmission. Our results, which also capitalized on publicly available assemblies, expand the range of *Spiroplasma* lineages that encode a particular combination of toxin types and revealed the existence of a highly divergent lineage of *Spiroplasma* that associates with insects.

## Introduction

Heritable associations between insects and bacteria are pervasive, influential and taxonomically and functionally diverse [1]. Whereas a small proportion of insects engage in obligate (i.e. absolutely necessary for the host) mutualisms with heritable bacteria, over half of the insect species are estimated to harbour facultative (i.e. non-essential) bacteria, whose predominant mode of transmission is via maternal transfer [1,3]. The impact of these heritable facultative bacteria, the most common of which is the genus *Wolbachia* (*Alphaproteobacteria*; intracellular), is far-reaching and ranges from detrimental to beneficial phenotypes, including manipulation of host reproduction (male killing, cytoplasmic incompatibility, parthenogenesis induction and feminization [4], as well as increased and reduced susceptibility to environmental stressors and natural enemies (reviewed by [5]). Some of these phenotypes are being exploited for solving major human challenges, such as the use of *Wolbachia* to reduce dengue virus transmission by *Aedes* mosquitoes [6]. Because most heritable bacteria are practically impossible to culture outside the host (for some exceptions, see [7]), comparative and functional genomics tools have proven invaluable for uncovering the mechanistic basis and evolutionary history of such phenotypes, particularly when hypothesized mechanisms can be further queried with the genetic tools afforded by model insects (e.g. the evolution and mechanistic basis of cytoplasmic incompatibility in *Wolbachia* [8,11]). Substantial research progress has also been achieved with other influential heritable bacteria of insects, including the Gammaproteobacterium *Hamiltonella* of aphids (e.g. [12,14]), and members of the class *Mollicutes* genus *Spiroplasma*.

*Spiroplasma* are small cell-wall-less bacteria strictly dependent on eukaryotic hosts, commonly arthropods and plants, with which they form pathogenic (e.g. the insect-vectored plant pathogens *Spiroplasma citri* and *Spiroplasma kunkelii*), commensalistic or mutualistic associations [15]. *Spiroplasma* are estimated to infect up to 7% of terrestrial arthropod species [16]. *Spiroplasma* inhabit both intra- and extra-cellular (e.g. insect haemolymph) environments. Under certain conditions/environments, many are motile and acquire the helical (spiral) shape implied by their name [15]. It was these features that revealed their presence in drops of *Drosophila* haemolymph observed under the microscope to early researchers, who initially thought they were spirochaetes [17]. The genus is composed of three large clades: Citri+Poulsonii+Chrysopicola+Mirum, Ixodetis and Apis [18]. The Apis clade appears to lack vertically transmitted members, whereas the other two clades contain both vertically and horizontally transmitted members [19 and references therein]. Like other vertically transmitted endosymbionts, heritable *Spiroplasma* are fastidious [7,20, 21]. Whilst some progress has been made towards *in vitro* culture and transformation [22,23], heritable *Spiroplasma* are not genetically tractable in practical terms. Nonetheless, the common association of heritable *Spiroplasma* with tractable insect hosts (e.g. several members of *Drosophila*, aphids and coccinellid beetles) has contributed to their establishment as a valuable system for the study of insect-bacteria interactions [24,25].

Approximately 20 species of *Drosophila*, representing divergent clades, are known to naturally host *Spiroplasma* [26,27, reviewed by 28]. Members of three major *Spiroplasma* clades associate with *Drosophila*. Transgenerational transmission, interpreted as evidence of vertical transmission, has been demonstrated for *Drosophila-Spiroplasma* associations that have been assessed (reviewed by [19,28]). Transovarial transmission, requiring *Spiroplasma* invasion of developing eggs from the haemolymph, has been demonstrated in the strain that naturally associates with the model organism *Drosophila melanogaster* [29]. However, lack of congruence between host and symbiont phylogenies reveals multiple instances of horizontal acquisition of *Spiroplasma* by *Drosophila* [30,31], and interspecific horizontal transmission via ectoparasitic mites, which are commonly found on wild *Drosophila*, has been demonstrated in the lab [32]. The fitness consequences of *Spiroplasma* infection for *Drosophila* are diverse: (a) neutral (or unknown); (b) beneficial in certain contexts, such as enhanced tolerance or resistance against particular natural enemies that include endo-macroparasites (parasitic wasps and nematodes) (e.g. [33,34]), as well as against bacteria and fungi [35]; (c) reproductive manipulation in the form of male killing (reviewed in [25]); and (d) detrimental in the form of shortened life span [36] or increased susceptibility to certain pathogens [37].

Most knowledge on the *Drosophila-Spiroplasma* association has been gleaned from the Poulsonii clade, which (a) contains male-killing (referred to as ‘SRO’=sex ratio organisms, in early studies) and non-male-killing strains of *Drosophila*, (b) achieves relatively high densities within individual hosts [38], (c) exhibits relatively high vertical transmission fidelities and (d) includes all of the known defensive *Spiroplasma* of *Drosophila* (with the exception of an unpublished report from the Ixodetis clade associated with *Drosophila atripex* [19]). Whilst male-killing strains of the Poulsonii clade tend to achieve low prevalence in wild populations, non-male-killing strains can achieve high prevalence (reviewed by [28]), likely due to their defensive phenotype (e.g. [33]). Substitution rates in the Poulsonii clade are amongst the highest reported for any bacteria [39], a feature that likely explains the repeated loss of the male-killing phenotype in lab populations. Poulsonii also contains the only successfully *in vitro* cultured *Drosophila*-associated *Spiroplasma*, which facilitated the initial comparative/functional genomics and proteomics studies [23,40,42]. With the aid of the heterologous expression tools provided by *D. melanogaster*, and to a lesser extent by *Escherichia coli* [43], much has been elucidated about the host and *Spiroplasma* factors and mechanisms involved in the male-killing and wasp/nematode-killing mechanisms [44,51]. In both phenotypes, *Spiroplasma*-encoded toxins, including ribosome-inactivating proteins (RIPs; similar to ricin and Shiga toxin), ovarian tumour deubiquitinases (OTUs) and/or ankyrin repeats, are implicated (see the ‘Results and discussion’ section).

The Poulsonii clade is associated with members of the two major groups of *Drosophila* (i.e. subgenus *Sophophora*, which contains *D. melanogaster*, and subgenus *Drosophila*). In contrast, the much less studied *Drosophila*-associated Citri clade (a) is restricted to members of *repleta* [27,30, 31, 52], a subgenus *Drosophila* group that contains most cactophilic *Drosophila* [53,54]; (b) exhibits comparatively lower titres [38]; (c) achieves lower vertical transmission fidelity, based on its frequent loss from lab cultures of its hosts; (d) is not known to kill males; and (e) can exhibit a range of infection frequencies in wild host populations (up to 85%; [52].

Based on sequences of the 16S rDNA gene, three distinct *Drosophila*-associated Citri clade strains were previously known [30,31]: (1) *s*Moj in *Drosophila mojavensis*; (2) *s*Ald-West (originally *s*Ald) in *Drosophila aldrichi* from Tucson, Arizona, and in *Drosophila wheeleri* from Catalina Island, California; and (3) *s*Hy2 (= *s*Hyd2) in *Drosophila hydei*. *D. hydei* is also host to the Poulsonii clade strain *s*Hy1 (= *s*Hy, *s*Hyd or *s*Hyd1 in other studies), a strain that is geographically widespread, including Great Britain and Japan [30,31, 52, 55,58], and whose genome was recently reported [39]. Both *s*Hy1 and *s*Hy2 co-occur in several localities in the American continent (Arizona and the Mexican states of Oaxaca, Sonora and Estado de Mexico), which is the native range of *D. hydei* [54], but have not been recorded within the same individual fly [27,30, 31, 52]. Co-occurrence within the same individual host would provide an arena for gene exchange between the two *Spiroplasma* strains.

Here, we report the discovery and infection prevalence of *s*Ald-Tx (a fourth *Drosophila*-associated Citri clade strain), detected in two sympatric (non-sister) species of cactophilic *Drosophila* (*Drosophila mulleri* and *D. aldrichi*) in the Texas Hill Country region. We also report on the fitness consequences of its close relative *s*Moj (native to *D. mojavensis*) in the context of wasp parasitism. We use short reads (Illumina) from whole-genome shotgun (WGS) DNA libraries to assemble, annotate and compare the draft genomes of sAld-Tx, *s*Moj and their close relative *s*Hy2 (native to *D. hydei*). We perform phylogenomic analyses of available representatives of the Citri and Poulsonii clades, along with outgroup taxa. We compare the inferred metabolic and DNA repair capacities between the Citri and Poulsonii clade *Drosophila*-associated strains and their close relatives. Finally, we analyse putative toxin genes in our target strains, as well as in recently released genome assemblies of numerous *Spiroplasma* strains (most reflect byproducts of genome projects aimed at their insect hosts), including what appears to be a new clade of *Spiroplasma* associated with insects (termed ‘clade X’ pending a more formal assessment).

## Methods

### *Spiroplasma* taxon naming convention

Because most of the *Spiroplasma* species or strains referred to in this study have not been formally described, we adopt the common practice, which has also been used for *Wolbachia*, of referring to unnamed *Spiroplasma* strains/species by lower case ‘*s*’ (for *Spiroplasma*) followed by the first few letters of their host’s species name (e.g ‘*s*Mel’ for the *Spiroplasma* strain of *D. melanogaster*). In some cases, we add a number or region identifier to such labels.

### Specimen sources

We used banana+yeast baits or sweep-netting over a compost to collect wild *Drosophila* and parasitic wasps at several locations in the Austin, San Marcos and San Antonio areas of Texas (for *D. aldrichi* and *D. mulleri*; distinguished on the basis of the coloration pattern of the tergites; https://flybase.org/reports/FBim0000512.html and https://flybase.org/reports/FBim0000511.html [59]); at Catalina Island, California (for *D. mojavensis*); and in central Mexico (for *D. hydei*). Several of the wild-caught females were used to establish isofemale lines; one per each of *D. aldrichi*, *D. mojavensis* and *D. hydei* was used to sequence the genomes of their naturally occurring Citri clade *Spiroplasma* strains (Table S1, available in the online Supplementary Material). All insects were maintained on banana-Opuntia food (Protocol S3) at 25 °C (12 : 12 light:dark cycle).

To determine whether flies were infected with *Spiroplasma*, DNA extractions of individual whole flies were subjected to PCR with the *Spiroplasma*-specific primers 16STF1 and 16STR1 [30] with annealing settings of touchdown 65–55 °C, which target an ~1,368 bp region of the 16S rRNA gene. A subset of *Spiroplasma*-positive samples was subjected to Sanger sequencing. To estimate the prevalence of *Spiroplasma* in time and space (in *D. aldrichi* and *D. mulleri* from Texas), we counted the number of positive and negative individuals. DNA extractions that did not produce amplicons with the *Spiroplasma*-specific PCR were subjected to PCR of the host-specific mitochondrial gene cytochrome oxidase I (COI) with primers HCO2198 and LCO1490 [60]. Extractions that yielded no COI amplicon were deemed of inadequate DNA quality and thus excluded. All PCRs included positive (template from known *Spiroplasma*-infected fly) and negative (no DNA template) controls. Because *D. hydei* is the natural host of a Citri clade (*s*Hy2) and a Poulsonii clade (*s*Hy1) *Spiroplasma* [31], positive 16STF1-16STR1 amplicons of *D. hydei* individuals were subjected to separate restriction digestion reactions, each containing a different enzyme that targets diagnostic positions between the two strains (Table S2).

Three DNA isolation methods were used. For *Spiroplasma* screening purposes, we used ‘squish prep’ [61] to extract DNA from individual flies. For WGS Illumina sequencing, 1–2 g of whole adult flies (~1–2 weeks old) from *Spiroplasma*-positive *D. hydei* and *D. aldrichi* (Table S1) was collected for separate Cetyltrimethylammonium Bromide (CTAB)-phenol-based DNA extractions (Protocol S1).

DNA from *s*Moj-infected *D. mojavensis* was isolated with a chloroform-ethanol extraction protocol (Protocol S2) from haemolymph obtained by piercing the mesothoracic segment of ~300 individuals belonging to the infected isoline CI-33-15. Immediately after the piercing, ~35–40 flies were placed into 0.5 ml microcentrifuge tubes previously pierced in the bottom, which were placed within a 1.5 ml microcentrifuge tube containing ~20 μl PBS solution 1X (PBS buffer; 137 mM NaCl, 2.7 mM KCl, 10 mM Na2HPO4 and 1.8 mM KH2PO4), and centrifuged at 7,000 r.p.m. (g=4.5) for 10 s.

### Fitness assays to evaluate the effect of *Spiroplasma s*Moj on *D. mojavensis*, in the context of parasitism by two wasps

We used the following procedures to evaluate the effect of *Spiroplasma s*Moj on larva-to-adult survivorship of * D. mojavensis* in the context of wasp parasitism. We used *D. mojavensis* isoline CI-33-15 (Table S1) to establish *Spiroplasma*-infected and *Spiroplasma*-free sub-isolines (i.e. those that naturally lost the infection). Flies were allowed to oviposit on Opuntia-banana medium for 48 h and transferred to a new vial for a second oviposition, after which they were removed and subjected to individual DNA extraction (squish-prep) and *Spiroplasma*-specific PCR with 16STF1 and 16STR1 primers to verify infection status. For the *Spiroplasma*-infected treatment, only vials (replicates) in which all such females were *Spiroplasma*-positive were retained. Subsequently, 30 second-instar larvae were collected and transferred to a new vial (i.e. replicate), where they were subjected to one of the following wasp treatments: no wasp control, *Leptopilina heterotoma* Lh14 and *Asobara* sp. w35 (Table S1). Those subjected to wasps were exposed to adult female wasps in a 1 : 6 wasp:larvae ratio for 24 h. Fifteen replicates for each of the six combined treatments (i.e. *Spiroplasma* x wasp) were obtained (see the ‘Results and discussion’ section). Values of initial larvae, puparia, eclosing adult flies and eclosing adult wasps were recorded.

**Table 1.** Assembly and annotation statistics for the three *Drosophila*-associated Citri clade strains, for *s*Fus (from *Glossina fuscipes*), and the previously reported *Drosophila*-associated Poulsonii clade (for comparison). For consistency, the number of CDS (coding) genes and pseudogenes is based on the latest RefSeq annotation when available. The contigs and annotation files for *s*Fus are provided in Dataset S24

	Citri clade			Poulsonii clade			
	***s*Ald-Tx**	***s*Hy2**	***s*Moj**	***s*Mel**	***s*Hy1**	***s*Neo**	***s*Fus**
BioProject	PRJNA506491	PRJNA506493	PRJNA355307	PRJNA256019	PRJNA224116	PRJNA492288	PRJNA172853
WGS accession no.	RXFY00000000	RXFZ02000000	MQTY00000000.2	SSBE00000000.1	NZ_CP093047.1–NZ_CP093052.1	RAHC00000000	JFJR01000000
Annotation accession no.	GCF_044714355.1	GCF_044714365.2	GCF_016082285.1	GCF_009866525.1	GCF_022569815.1	GCF_003989055.1	
Annotation name	GCF_044714355.1-RS_2024_11_09	GCF_044714365.1-RS_2025_04_14	GCF_016082285.1-RS_2025_02_23	GCF_009866525.1-RS_2024_08_05	GCF_022569815.1-RS_2024_12_16	GCF_003989055.1-RS_2025_02_25	Prokka
Contigs	202	384	174	21	6	181	87
Nucleotides	1,109,497	1,230,639	1,071,638	1,938,611	1,625,797	1,783,629	1,168,733
GC%	26.8	26.3	26.8	26.5	27.5	26.5	28.1
Max	42,261	23,369§	30,814				148,233
Min	202	506	544				725
Contig N50	8,940	5,515	9,256	144,800	1,625,797	97,866	30,388
Coverage (X)	27	336	105	122.7	30	214	120*
BUSCO completeness (*Mollicutes* 151)	98.7	98	98	97.4	91.4	98	98.7
BUSCO completeness (*Entomoplasmatales* 332)	94.3	92.8	93.4	94.9	87.6	96.4	97.3
CDS (coding)	1,133‡	1,260‡	985‡	2,175‡	1,706‡	2,279‡	1,275†
Pseudogenes	54‡	65‡	50‡	97‡	63‡	89‡	?†
rRNA	3	3	3	3	3	3	3†
tRNA	32	32	31	31	30	31	32†

*Based on coverage reported for metagenome assembly

†based on Prokka annotation; pseudogenes not reported

‡based on RefSeq

§only one contig, the largest one in this assembly (i.e. RXFZ02000001; 23,369 bp), was effectively contributed by the MinION long-read dataset. A total of 1,082,116 MinION reads were assembled (combined length=1.2 Gbp; N50 length=49,474 bp). Based on a blastn search against the NCBI nt database (Oct 2018), only seven contigs (combined length=54,595 bp; assembled from 726 reads) had a hit to *Spiroplasma*, of which five were assembled into contig RXFZ02000001 (and corrected with Illumina reads), which shares similarity with phage-like contigs of *Drosophila*-associated Poulsonii clade *Spiroplasma* (see the ‘Results and discussion’ section; Fig. S5). The remaining two MinION-based contigs, which appeared to have low quality and coverage, were encompassed by longer contigs that were assembled based on Illumina-only reads.

Graphing of results and statistical analyses were performed with the statistical software R, version 4.1.2 (R Development Core Team 2018). Fly and wasp survival measures were analysed by fitting a generalized linear model with a binomial distribution (or quasibinomial distribution when there was evidence of overdispersion). Each wasp treatment was analysed separately. The significance of the independent variable *Spiroplasma* was assessed with the analysis of deviance table (type II tests), as implemented in the ‘car’ package. Raw data (Dataset S1) and R command lines used (Command Line S1) are available in Figshare.

### Preparation and sequencing of DNA libraries

Illumina libraries of DNA extractions from *s*Moj-infected *D. mojavensis*, *s*Ald-Tx-infected *D. aldrichi* and *s*Hy2-infected * D. hydei* were submitted to the Texas AgriLife Genomics and Bioinformatics Services Facility (College Station, TX) for library preparation (WGS; Illumina) and sequencing. The *s*Moj library (paired-end 100 bp) was sequenced on the HiSeq^®^ 2500 Sequencing System (Illumina, Inc.), whereas the *s*Ald-Tx and *s*Hy2 libraries (paired-end 150 bp) were sequenced on the NovaSeq 6000 system within an S2 flowcell (Illumina, Inc.).

In an attempt to enhance the assembly of strain *s*Hy2, we used the following protocols to obtain long reads on the MinION platform (Oxford Nanopore v2.1). To attempt enrichment of *Spiroplasma*, we collected haemolymph from *s*Hy2-infected * D. hydei* (H25 strain) and passed it through a 70 µm filter. DNA was isolated from the filtered haemolymph using a chloroform-ethanol protocol (Protocol S2). Because the amount of DNA was insufficient for the library preparation protocol, we combined it with (‘spiked it into’) a DNA sample from the tephritid fruit fly *Anastrepha striata* (confirmed to be *Spiroplasma*-free). The combined DNA was prepared for Oxford Nanopore sequencing with the Nanopore SQK-LSK109 sequencing kit following the manufacturer’s recommendations. Nanopore was run on a Spot-on flow cell Mk 1 R9 (FLO-MIN106), MinION Mk1B sequencer, utilizing the Nanopore sequencing software MinKNOW v2.1 (Oxford Nanopore). Basecalling was performed with Albacore v2.3.1 (Oxford Nanopore), and adapter sequences were removed from resulting reads with PoreChop (https://github.com/rrwick/Porechop).

### Genome assembly

Illumina raw data were inspected for quality with FastQC [62]. Then, Trimmomatic v.0.39 [63] was used to remove adapter sequences and filter low-quality sequences using default parameters. To remove non-target (i.e. non-*Spiroplasma*) host reads, each library was aligned with Bowtie 2 v.2.3.2 [64] to the closest *Drosophila* genome assembly available at the time (lacking *Spiroplasma* infection): *D. hydei* ASM278046v1 for *D. hydei* and *D. mojavensis* dmoj_caf1 for *D. aldrichi* and *D. mojavensis*. Unmapped reads from the *D. hydei*, *D. aldrichi* and *D. mojavensis* libraries were separately subjected to assembly with SPAdes v.3.12 [65]. Geneious Prime 2020.2.2 (Biomatters Ltd.) was used to obtain a single consensus *de novo* assembly per library (using default parameters).

#### Final assembly quality control

To reduce the inclusion of chimaeric contigs and assembly artefacts in the final assemblies of each library, a series of quality control steps were applied to the metagenomic sequencing data. Assembled contigs with a length of 200 bp or more were subjected to blastn [66] to the NCBI nt database (Oct 2018). Contigs with high similarity (E-value=1E^−50, PI>20) to *Spiroplasma* or closely related taxa (i.e. members of class *Mollicutes*) were considered to belong to the target *Spiroplasma* strain and retained. The reads were aligned to these selected contigs with Bowtie2 v.2.3.2 (default parameters), and the results were visualized and processed in Geneious. Contigs containing a region (excluding repeat regions located in the middle of the contig) with coverage below a certain threshold (30× for *s*Hy2; 15× for *s*Ald-Tx and *s*Moj) were then broken apart at that site via the ‘Generate Consensus Sequence’ option of Geneious. Nucleotide locations composed of more than one type of base were called as base a if that base was in at least 50% of the reads mapped to that position. Otherwise, the consensus was assigned the corresponding IUPAC ambiguity code at that position.

To search for potentially missing regions in each of the three assemblies (*s*Ald-Tx, *s*Hy2 and *s*Moj), we performed the following complementary approaches (see Protocol S4). We used LASTZ v.1.04.15 (Large-Scale Genome Alignment Tool) [67,68] to align our three genome assemblies to *Spiroplasma phoeniceum* and *S. kunkelii* (i.e. their two closest relatives whose assemblies include a whole chromosome), and to *Spiroplasma melliferum* (a more distant relative that has comparatively greater metabolic capacities). We then used our short reads mapped to the same reference assemblies and to our other two assemblies (e.g. *s*Hy2 reads to *s*Moj and *s*Ald-Tx assemblies; see Protocol S4), to determine whether such missing regions had coverage by short reads. If so, the short reads mapped to those regions were extracted and subjected to *de novo* assembly within Geneious (details in Protocol S4). The resulting contigs are considered to be the final draft assembly for *s*Ald-Tx and *s*Moj.

The MinION trimmed fastq output (*s*Hy2) was assembled with the long-read assembler CANU [69]. The resulting contigs were then subjected to a blastn search (as described above). CANU (long-read) contigs with high similarity to *Spiroplasma* were retained and used to augment (or merge with) the *s*Hy2 (short-read) SPAdes assembly using Geneious Mapper (details on the number of long reads and contigs are in Table 1). The resulting assembly was then used as a reference to map the *s*Hy2 Illumina reads with Geneious Mapper (5× iterations) to correct MinION-induced indels. The resulting consensus contigs represent the final draft assembly for *s*Hy2.

### Annotation

*s*Ald-Tx, *s*Hy2 and *s*Moj genome assemblies were submitted to the NCBI Prokaryotic Genome Annotation Pipeline (PGAP) pipeline to obtain final annotations (Table 1). We used the following additional annotation tools. Protein sequences for the *Spiroplasma* genomes were annotated using the BlastKOALA KEGG tool v.2.2 (September 2019 [70]). The generated KEGG (Kyoto Encyclopedia of Genes and Genomes) Orthology (KO) numbers were then compared amongst several *Spiroplasma* genomes to determine differences in metabolism and DNA repair. Additionally, protein sequences were annotated for Cluster of Orthologous Groups (COG) by EGGNog v.5.0 [71]. To assess completeness, the NCBI PGAP protein sequences were subjected to BUSCO (Benchmarking Universal Single-Copy Orthologs) v.5.5.0 analyses, as implemented in the usegalaxy.eu server (mode=prot; lineage data source=cached database with lineage all +2024-03-21-114020; lineage=*Mollicutes* and *Entomoplasmatales*). Genes with KOs of interest were compared via blastn to a nucleotide database composed exclusively of *Spiroplasma* strains (Table S3), to confirm their presence/absence in the Citri and Poulsonii clades. KO numbers and BUSCO *Entomoplasmatales* genes present in the Citri clade assemblies deemed as reference (i.e. *S. phoeniceum*, *S. kunkelii* and/or *S. melliferum*), but absent (missing, fragmented and pseudogenized) in one or more of our three assemblies, were further scrutinized with the procedures described above aimed at finding and assembling potentially missing regions.

In the process of annotating these genomes, we discovered and extracted 87 contigs (Table S4) in the draft genome assembly of the tsetse fly *G. fuscipes fuscipes* (PRJNA172853, WGS JFJR00000000.1) that we assigned to *Spiroplasma* on the basis of blastn [NCBI nt database (Oct 2018)]. We also learnt of the recent release of numerous *Spiroplasma* genome assemblies obtained from sequencing projects aimed at their arthropod hosts, many of which encode putative toxin genes (e.g. search for ‘spiroplasma [organism] AND inactivating’ at https://www.ncbi.nlm.nih.gov/protein). The majority of these genomes had gene annotations available in NCBI. A preliminary phylogenetic analysis of the 16S ribosomal RNA gene allowed us to identify the major *Spiroplasma* clades to which they belong (Fig. S1 and Dataset S2). A small number of these genomes fell within the Citri and Poulsonii clades and were thus included in the phylogenomic analyses. Because most of the remaining genomes fell within more distantly related clades (e.g. Apis, Ixodetis and a previously unknown clade hereafter referred to Clade X), they were not included in the phylogenomics analyses and were only partially examined for other features of interest (e.g. toxin genes). Establishing the phylogenetic position of Clade X is beyond the scope of this study, but based on the 16S rDNA gene (Fig. S1), it belongs to the Spiroplasma–Entomoplasmataceae–Mycoides clade sensu [72].

We used Prokka v.1.14 [73] assuming the genetic code of *Mycoplasma*/*Spiroplasma* (i.e., translation table 4) implemented within the Galaxy Project platform [74] to identify all putative protein-coding regions in genome assemblies lacking publicly available gene annotations and in all Citri and Poulsonii clade genomes used in the phylogenomics analyses. Representatives of a sister lineage (the Chrysopicola clade), as well as the outgroup (Mirum clade), were also included (Dataset S3). Predicted protein sequences were analysed with InterPro v. 5.25 [75,76] to identify putative domains, implemented in usegalaxy.eu and https://www.ebi.ac.uk/interpro/ servers, using all the ‘Member databases’ and ‘Other sequence features’ available with their preconfigured cut-off thresholds.

To search for genes encoding potential toxins, we searched the annotations (InterPro, Koala and NCBI) for the following terms, most of which reflect toxins found in *Spiroplasma* or in other arthropod endosymbionts (reviewed in [77]): ‘toxin’; ‘lethal’; ‘inactivating’ and ‘ricin’ (for RIP); ‘etx’, ‘mtx’, ‘pore’ (for certain pore-forming proteins) and ‘ankyr’ (for ankyrin); ‘fic’ (for filamentation induced by cAMP), ‘adp’, ‘protective’ and ‘antigen’ [for ADP-ribosyltransferase exoenzyme and its associated protective antigen (PA)]; ‘cdt’ (for the cytolethal distending toxin); ‘cif’ (for the cytoplasmic incompatibility factor); ‘pqq’ (for PQQ-binding-like beta-propeller repeat protein); and ‘latro’ (for latrotoxin). Genes with annotations associated with potential toxins were subjected to further analysis to infer their evolutionary history and potential function. A subset of inferred protein products was determined to be truncated at the N or C termini, as they only contained single domains that are usually found within larger multi-domain proteins. To determine if a putative gene of interest was broken up by a missense mutation causing truncated single-domain gene products, we searched for the ‘missing’ regions/domains in the immediate flanking regions on their 5′ and 3′ ends. Similarly, because of the fractured nature of the assemblies in the Citri clade strains *s*Moj, *s*Ald-Tx and *s*Hy2, and the detection of intact and pseudogenized transposases in several contigs (see the 'Results and discussion' section), we also searched for potentially missing domains of genes in other contigs (e.g. in cases where the end of a contig occurred within a predicted partial protein of interest).

Because a search for the term ‘inactivating’ in the gene annotations revealed the presence of RIP genes of eight out of nine Clade X strains, and several of these genes shared substantial similarity with those of clades Citri and Poulsonii, we analysed Clade X genome assemblies with Prokka and InterProScan to find all predicted proteins with domains of interest. For the Ixodetis and Apis clades, we extracted the genes of interest based on term search in their NCBI gene annotations only.

For certain genes of interest, such as toxins, we extracted specific domain regions from the amino acid sequences, aligned them with the MAFFT v.7.490 [78,79] plugin of Geneious and performed phylogenetic analyses with IQTREE 2.2.2.6 COVID-edition for Mac OS X 64-bit built on 27 May 2023 [80,82]. For a subset of genes and taxa, we also performed phylogenetic analyses on the nucleotide sequences (see the ‘Results and discussion’ section).

Strains for which the term ‘crispr’ and/or ‘cas9’ was detected in their annotation were analysed with CRISPRCasTyper v1.8.0 [83], as implemented in the web server (https://crisprcastyper.crispr.dk/; accessed 15 July 2024; default settings).

To further examine genes of potential plectroviral origin, we used WP_339038695.1 (product=plectrovirus svts2 rep protein) as a query for a Delta-blast search [84] against NCBI’s non-redundant database.

### Phylogenomic analyses

To identify single-copy orthologs, the Prokka-derived amino acid sequences of 35 genomes (Dataset S3) were analysed with OrthoFinder2 v.2.5.5 [85] (default parameters) and subsequently aligned with MAFFT v7.471 (-l-INS-I, with the -phylipout option). Gene loci alignments were assessed for recombination with PhiPack [86], utilizing windows of 10, 20, 30, 40 and 50 amino acids. Gene loci with a *P*-value of 0.05 or less with one or more of the window sizes were considered to have significant recombination and removed from further analysis. The remaining genes were concatenated and subjected to maximum likelihood phylogenetic analysis with IQ-TREE. Trees from these and the remaining analyses were visualized and edited in FigTree v1.4.4 (http://github.com/rambaut/figtree/). Trees and other figures were further edited in Inkscape (https://inkscape.org/).

## Results and discussion

### Identity and infection frequencies of a newly discovered *Spiroplasma* strain (*s*Ald-Tx) in wild populations of * D. aldrichi* and *D. mulleri* from Texas

The 16S rDNA sequences of *Spiroplasma* from *D. aldrichi* and *D. mulleri* from Texas were identical to each other and were (1 out of 973; or 11 out of 1,293 bp=0.85% uncorrected p distance) different from the strain previously reported in *D. aldrichi* and * D. wheeleri* from California (*s*Ald-West; GenBank Acc. nos. FJ657236 and FJ657227; Fig. S2 and Dataset S4). Hereafter, we refer to the *Spiroplasma* strain associated with *D. aldrichi* (and *D. mulleri*) from Texas as *s*Ald-Tx (or *s*Ald-East).

The infection prevalence of *s*Ald-Tx varied broadly (4–94%) across its two host species, sites and years, but infection prevalence was consistently higher in *D. aldrichi* compared to *D. mulleri* (overall 71 vs. 38%, respectively; Table S5). These infection frequencies are within the range reported for other *Drosophila*-associated Citri clade strains: 33 and 85% in *D. mojavensis* (Arizona and California, respectively), 5.5% in *D. aldrichi* from Arizona and 53% in *D. wheeleri*. *s*Hy2 prevalence values in *D. hydei* are unknown, as most studies only detected *s*Hy1 [55,58] or did not separate *s*Hy1 from *s*Hy2 when computing *Spiroplasma* frequencies [52]. The highest reported *Drosophila*-associated *Spiroplasma* prevalence is that of *s*Neo (Poulsonii clade) in certain populations of *Drosophila neotestacea*, where substantial fitness benefits in the form of protection against nematodes and parasitoid wasps, and no reproductive manipulation has been reported [33,87]. In contrast, the infection frequencies of *Drosophila*-associated male-killing *Spiroplasma* strains tend to be <5% [17,88,90].

### No evidence of fitness benefits from *Drosophila*-associated Citri clade *Spiroplasma*

**Fig. 1.**
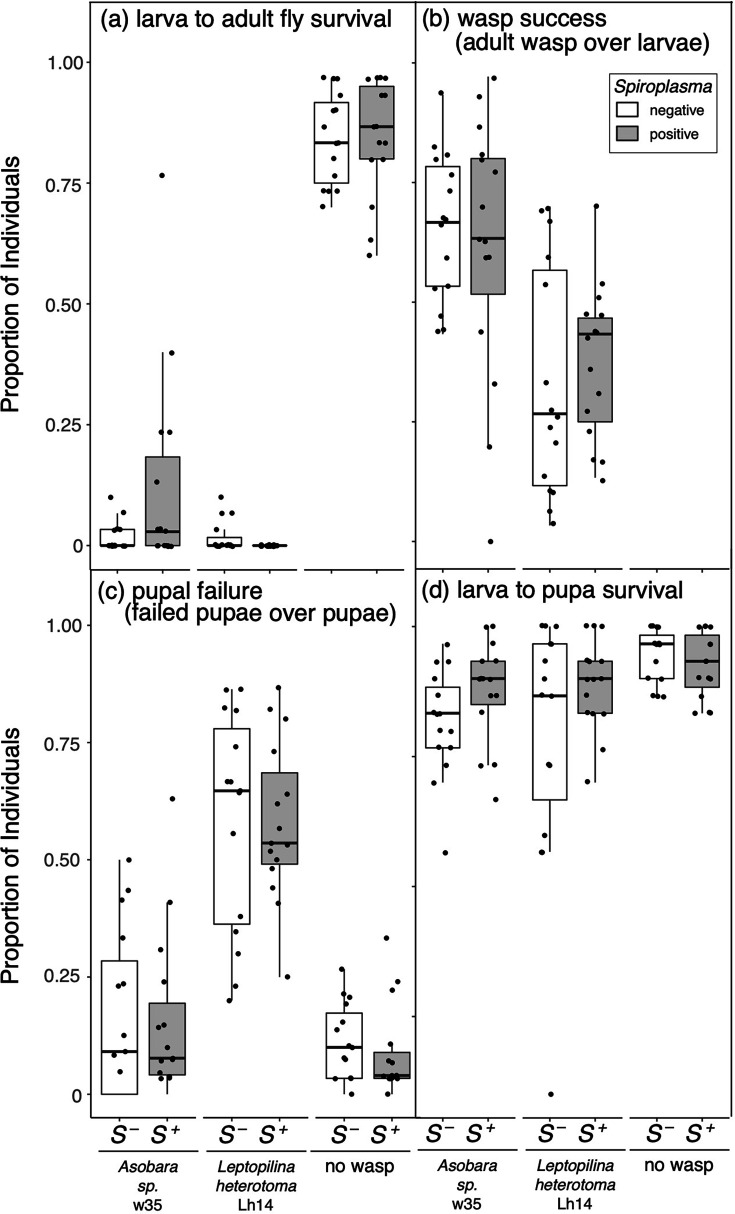
Effect of *Spiroplasma s*Moj (in *D. mojavensis*) on selected fly and wasp success and failure measures: (**a**) larva to adult fly survival, (**b**) wasp success, (**c**) pupal failure and (**d**) larva to pupa survival. None of the comparisons were significant. Points represent each measurement obtained. Box plots display the median, upper and lower quartiles, and the range excluding points beyond 1.5 × inter-quartile range. Grey boxes indicate *Spiroplasma*-infected (*S*^+^); white boxes indicate *Spiroplasma*-free (*S*^–^). Wasp treatments (*Asobara* sp. w35, *L. heterotoma* Lh14 and no wasp control) are indicated in the X-axis.

Our results revealed that *s*Moj does not significantly affect the larva-to-adult survivorship of its native host *D. mojavensis* following exposure to one of two divergent parasitic wasps (Fig. 1a, c and d and Table S6): the braconid Aw35 and the generalist figitid *L. heterotoma* (strain Lh14). Strain *s*Moj also did not significantly affect the success of these wasps developing in *D. mojavensis* (Fig. 1b and Table S6). In contrast, wasp Lh14 is highly susceptible to *Spiroplasma* from the Poulsonii clade in several different *Drosophila* species [34,87, 91,94]. Wasp Aw35 has not been tested against Poulsonii clade strains. Similarly to *s*Moj, Martinez-Montoya [27] reported that *s*Ald-Tx in *D. aldrichi* does not offer protection against wasps Aw35 and Lh14. The effect of *s*Hy2 on *D. hydei*, and of *s*Ald-Tx in its other native host *D. mulleri*, in the context of wasp exposure has not been established. It is possible that these Citri clade strains protect against other natural enemies, particularly in light of their encoding of several putative toxin genes (see below). There is a huge known and predicted diversity of *Drosophila* parasitoids [95], of which only approximately ten species have been assessed for susceptibility to one or more *Spiroplasma* strains [27, 96 and references therein 97]. Our single attempt to sample *Drosophila* parasitoids in Catalina Island, California, where *s*Moj achieves high prevalence, failed, but several *Drosophila* parasitoid species, including Aw35, have been sampled in the habitat of *D. aldrichi* and *D. mulleri* in Texas [95]. In addition, it is possible that the Citri clade strains studied herein protect against other types of natural enemies (e.g. bacteria, viruses, protozoans and fungi), as enhanced host fitness against pathogenic fungi and/or bacteria has been documented for other *Spiroplasma* clades [35,98].

In the absence of wasps, Citri clade *Spiroplasma* strains *s*Moj and *s*Ald-Tx have weak to no effect on larva-to-adult fly fitness (Fig. 1 [27]). Reproductive phenotypes have been investigated for *s*Moj and *s*Hy2 [27], which ruled out cytoplasmic incompatibility in *s*Moj and found that *s*Moj-infected *D. mojavensis* and *s*Hy2-infected *D. hydei* tend to lay more eggs earlier than their *Spiroplasma*-free counterparts. In contrast, the Poulsonii clade strain *s*Hy1 does not exert a detectable effect on *D. hydei* oviposition [99]. Increased early oviposition was reported for the Poulsonii clade (male-killing) strain (WSRO) harboured by *Drosophila willistoni* [100]. An early mating propensity induced by the Poulsonii clade strain (NSRO) harboured by *D. nebulosa* was reported by Malogolowkin-Cohen and Rodrigues-Pereira [101]. No further fitness consequences of Citri clade strains on *Drosophila* have been examined. Unfortunately, experimentation with Citri clade strains has been more challenging than that with Poulsonii clade strains, due to the frequent unintentional loss within their native hosts, and the difficulties of artificially transferring and maintaining Citri clade strains over several generations [102 and personal observation]. Such difficulties might stem from the comparatively lower titres of the Citri clade (*s*Moj and *s*Hy2) vs. Poulsonii clade (sHy1 and the male-killer sMel [38]). How *s*Ald-Tx and *s*Moj strains can achieve relatively high infection frequencies in wild populations despite low vertical transmission efficiency and no evidence of net fitness benefits remains unknown and could rely on substantial horizontal transfer.

### Phylogenomic relationships

**Fig. 2.**
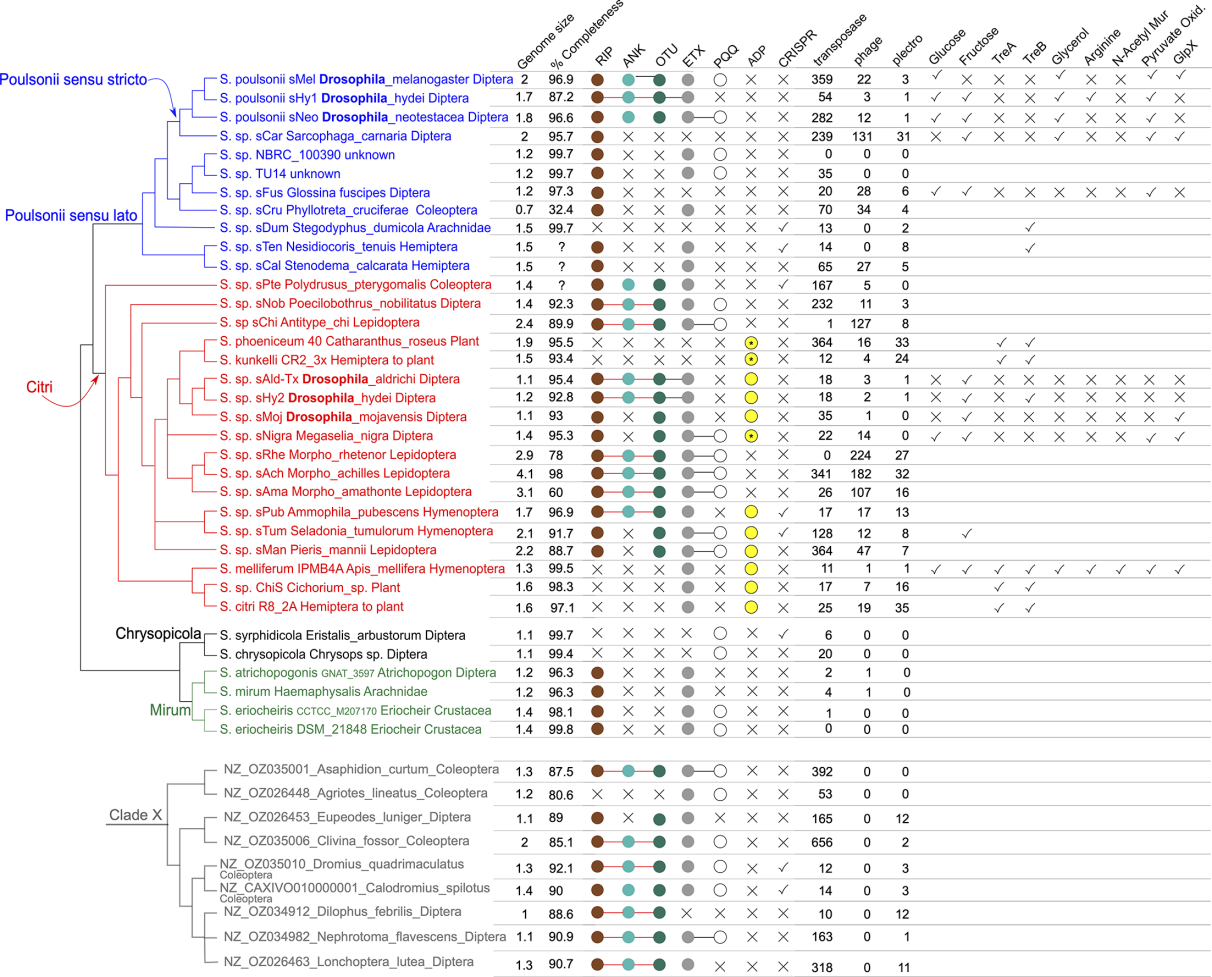
Cladogram depicting phylogenomic relationships of members of the Citri and Poulsonii clades and outgroups, and 16S rDNA phylogeny of members of Clade X. Phylogenetic distribution of genes of interest. The tree is based on the 62,325 amino acid sites from 189 single-copy genes with no evidence of recombination, except for the position of *s*Cru, *s*Rhe and *s*Ama (inferred based on a subset of 16,372 amino acid sites from 41 single-copy genes. A phylogram version including clade support values is provided in Fig. S19. Tip labels contain the *Spiroplasma* species or strain name, followed by the host Genus_species and its major taxonomic group. The Clade X tree (grey) is based on analysis of the 16S rDNA gene only. Genome sizes (Mb) and % completeness are based on what is reported on NCBI (RefSeq version when available). When available, completeness is based on CheckM analysis (v1.2.3) calculated on the PGAP gene set with the *Entomoplasmatales* CheckM marker set [150]; otherwise, it is based on our analysis with the BUSCO *Entomoplasmatales* set. Circles to the right indicate the presence of genes with domains annotated as RIP, ankyrin (ANK), OTU, ETX, PQQ and ADP (asterisks indicate that such gene is only found as pseudogenized). Circles connected by a single colour line indicate that they occur within a single CDS at least once in the corresponding genome. An X indicates the absence of such a gene. Columns with headers ‘transposase’, ‘phage’ and ‘plectro’ report the number of genes with such annotations. For the remaining columns, a check mark indicates presence, an X indicates absence and a blank indicates that its presence/absence was not determined.

We used phylogenomic analyses to infer the evolutionary history of currently available representatives of the Citri and Poulsonii clades plus outgroup taxa (Dataset S3). Three of the 35 genome assemblies targeted for our phylogenomics inferences (i.e. strains *s*Ama, *s*Rhe and *s*Cru) appeared substantially incomplete. Because their inclusion led to a small number (*n*=41) of single-copy orthologues, we excluded them from the main phylogenomics dataset, which encompassed 32 taxa and 189 genes lacking evidence of recombination. The concatenated alignments of the amino acid sequences of these 189 genes resulted in 62,325 positions, containing 33,279 distinct patterns and 24,622 parsimony-informative sites. The inferred tree (Fig. 2 and Dataset S5) reveals that the *s*Ald-Tx, *s*Hy2 and *s*Moj strains form a monophyletic group, with *s*Moj as sister to *s*Ald-Tx+*s*Hy2. This relationship is consistent with inferences from a smaller number of genes [30,31]. This relationship does not match the host phylogeny, because *D. mojavensis*, *D. mulleri* and *D. aldrichi* belong to the *mulleri* complex, which excludes *D. hydei* [103], implying horizontal transfer of *Spiroplasma* within this group of *Drosophila*. Additional evidence consistent with horizontal transmission includes the sharing of strain *s*Ald-Tx by sympatric specimens of *D. aldrichi* and *D. mulleri*, two species that are not sisters and are estimated to have diverged ~5.56 mya [103]. Whereas the sharing of strain *s*Ald-West by *D. wheeleri* from California and *D. aldrichi* from Arizona [30,52] could reflect another instance of horizontal transfer, taxonomic uncertainty includes the possibility that these two hosts are sister lineages whose common ancestor harboured *s*Ald-West [104,106]. Possible routes of horizontal transmission include via ingestion and vectored by ectoparasitic mites or parasitoid wasps. There are documented examples of different cactophilic *Drosophila* species sharing the same species of mites [107], of *Drosophila* mites harbouring *Spiroplasma* [108] and of interspecific transmission of *Spiroplasma* through mites [32]. Whereas no study has tested *Spiroplasma* transmission via parasitoid wasps, this transmission mechanism has been demonstrated for the haemolymph-dwelling symbionts *Hamiltonella defensa* and *Regiella insecticola* of aphids [109], and for *Wolbachia* in the whitefly *Bemisia tabaci* [110]. One study reports horizontal transmission of *Spiroplasma* (Poulsonii clade) in *Drosophila* via ingestion, but attempts to repeat this finding failed [17,100, 111].

The closest sister lineage to these *Drosophila*-associated Citri clade strains is the plant-pathogenic clade formed by *S. phoeniceum* and *S. kunkelii*. This *Drosophila*-associated plus plant pathogenic clade is joined at a polytomy with *s*Nigra (from the mushroom-feeding phorid fly *Megaselia* [112]), *s*Man (from the southern small white butterfly [113]), a clade of three *Morpho* butterfly associates (*s*Ach, *s*Rhe and *s*Ama [114]) and a clade associated with *Hymenoptera* (*s*Pub and *s*Tum). More distantly related strains associated with *Coleoptera* (*s*Pte), other *Diptera* (*s*Nob), other *Lepidoptera* (*s*Chi) and a clade formed by *S. citri*, *S. melliferum* and ChiS were recovered and collectively assigned to the Citri clade (red). Sister to Citri is what we refer to as the Poulsonii *sensu lato* clade (blue). Poulsonii *s. l*. contains a subclade composed of the previously characterized *Drosophila*-associated strains (*s*Mel, *s*Hy1 and *s*Neo). Sister to this *Drosophila*-associated clade is *s*Car, associated with another dipteran, the flesh fly *Sarcophaga carnaria*. This Poulsonii *sensu stricto* clade is sister to a clade containing previously characterized (and *in vitro* cultivated) strains from unknown hosts NBRC_100390 and TU14 [115,116] and two strains whose genomes have not been previously compared (*s*Fus from a tsetse fly and *s*Cru from a beetle). The most basal split in Poulsonii *s. l*. separates all of the above strains from two *Hemiptera*-associated strains (*s*Ten and *s*Cal).

### Genome assembly statistics and annotations

The assembly sizes of the three *Drosophila*-associated Citri clade strains (*s*Ald-Tx, *s*Moj and *s*Hy2) ranged from 1.07 to 1.23 Mb (Table 1), which is substantially smaller than those of their closely related plant pathogens (*S. kunkelii*: 1.5 Mb; *S. phoeniceum*: 2.2 Mb), and of the *Drosophila*-associated Poulsonii clade strains (1.6–1.9 Mb). The assemblies of *s*Ald-Tx, *s*Moj and *s*Hy2 were substantially fragmented, ranging from 174 (*s*Moj) to 384 (*s*Hy2) contigs, and contig N50 was small (5.5 kb in *s*Hy2 to 9.2 kb in *s*Moj), compared to that of Poulsonii clade genomes (0.98–1.6 Mb), which did not solely rely on short reads. The fragmented status of these assemblies precludes conclusive inferences about genome sizes, synteny and distribution and number of mobile/repetitive elements. RefSeq annotated 1,133, 1260 and 985 protein-coding sequences for *s*Ald-Tx, *s*Hy2 and *s*Moj, respectively (Table 1), which are substantially fewer than those in their Poulsonii clade counterparts (i.e. 1,706–2,279). Nonetheless, BUSCO completeness scores using the *Mollicutes* and *Entomoplasmatales* lineage databases (containing 151 and 332 groups, respectively) ranged from 98 to 98.7 and 92.8 to 94.3% (highest for sAld-Tx). The few genes that BUSCO classified as not ‘Complete’ (Table S9) were verified as missing or pseudogenized by examination of short-read mapping to the respective and/or the other reference assemblies (see Protocol S4). These BUSCO completeness scores are similar to most other assembled *Spiroplasma* genomes (e.g. [112,117, 118]). The number of tRNAs was similar amongst the assemblies (32 in *s*Ald-Tx and *s*Hy2; 31 in *s*Moj). Mean coverage was 27X for *s*Ald-Tx, 105X for *s*Moj and 336X for *s*Hy2. To minimize bias from misassembled repetitive chromosomal or extrachromosomal regions, mean coverage was measured on the present BUSCO genes from the *Entomoplasmatales* set (Table S9).

The assembly size of the Poulsonii *sensu lato* strain *s*Fus (from 87 contigs included with the *G. fuscipes fuscipes* assembly) was also relatively small (~1.17 Mb; Table 1), but not much smaller than the complete chromosome size (~1.2 Mb) of its sister lineage (comprised of strains TU-14 and NBRC_100390). Prokka annotated 1,275 protein-coding genes in *s*Fus, compared to 1,039 in TU-14 and NBRC_100390, but the number of pseudogenes in *s*Fus has not been determined (the contigs and annotation are provided in Dataset S24). BUSCO completeness scores for *s*Fus using the *Mollicutes* and *Entomoplasmatales* lineage databases were relatively high (98.7 and 97.3%, respectively).

#### Extrachromosomal and mobile genetic elements and the CRISPR/Cas system

Mobile genetic elements such as plasmids, phages and insertion sequence elements are common in numerous *Spiroplasma* genomes and other insect symbionts and contribute to the acquisition (and loss) of relevant functions (e.g. virulence factors) and rapid genome evolution [14,39, 77]. Whilst a full picture of such elements requires complete assemblies, which are lacking for the three *Drosophila*-associated Citri clade strains, below, we discuss preliminary inferences. It is unclear whether the three *Drosophila*-associated Citri clade strains contain plasmids, as our assemblies failed to recover circular contigs. However, the three assemblies contained contigs encoding a *ParA family protein*: one in *s*Moj and six in *s*Hy2 and *s*Ald-Tx, with one of each contig per strain also containing a *ParB* gene immediately downstream of the *ParA* gene. ParA and ParB are commonly involved in plasmid segregation [119]. Some of these contigs exhibit partial blast matches to known Citri and Poulsonii clade plasmids, but they also match parts of chromosomes (not shown). Concerning genes annotated as transposases, a substantially smaller number was detected in the *Drosophila*-associated Citri clade (*s*Ald-Tx=18; *s*Hy2=18; *s*Moj=35) than in its Poulsonii clade counterpart (*s*Mel=359; *s*Neo=282; *s*Hy1=54) (Fig. 2), but complete assemblies may reveal more.

Previous studies indicate that the Citri and Poulsonii clades lack a CRISPR/Cas system, suggesting that it was absent in their common ancestor [39,120]. However, our results from ccytper reveal a potentially functional CRISPR/Cas system in the following: (a) the Citri clade strain *s*Pte (Fig. S3 and Dataset S6), which is sister to the remaining members of the Citri clade (Fig. 2); (b) two Poulsonii clade strains (*s*Dum and *s*Ten) that are distantly related to those associated with *Drosophila* and the tsetse fly; and (c) two clade X strains. Although a functional CRISPR/Cas system is expected to prevent phage invasion/proliferation, all of these strains had at least two genes with a ‘phage’ or ‘plectro’ annotation. Similarly, as noted previously for TU-14 and NBRC_100390 [39], several strains lacking CRISPR/Cas were mostly/completely devoid of ‘phage’ and ‘plectro’ gene annotations (Fig. 2). The *Drosophila*-associated Citri and Poulsonii clade strains have very few ‘plectro’ genes (range 0–3), whereas several non-*Drosophila*-associated strains had >20 (e.g. *s*Car, *S. phoeniceum*, *S. kunkelii*, *s*Rhe, *s*Ach and *S. citri*; Fig. 2).

Further examination of sequences of potential plectroviral origin (i.e. Delta-blast search against the nr database using a ‘plectrovirus svts2 rep protein’ as a query) recovered genes found in *Spiroplasma* strains belonging to clades Ixodetis, Clade X, Apis (only one strain), Poulsonii and Citri. These sequences tended to show more similarity within clade (Fig. S4 and Dataset S7). No evidence of such proteins was found in the Citri clade strains *s*Ald-Tx, *s*Nigra, *s*Hy2 and *s*Moj (except for one short 54 aa ORF EHV01_0264). On the basis of the small number of genome assemblies at the time, Ku *et al*. [121] hypothesized that susceptibility to plectroviral invasion originated in the common ancestor of the Citri+Poulsonii (as defined herein) or in one of its subclades. Our results are in line with that hypothesis, if several independent losses are assumed, but the presence of sequences of plectroviral origin in the Apis and Ixodetis clades (Fig. S4) implies either additional independent invasion(s) or a single invasion in the ancestor of Citri+Poulsonii+Ixodetis+Apis, followed by multiple independent losses.

In addition to *Plectroviridae* (when active, characterized by non-enveloped rigid rods containing single-stranded DNA [122]), other types of particles and/or DNA sequences/features derived from phage occur in *Spiroplasma* (reviewed in [123,124]). Ramirez *et al*. [123] assembled phage-like contigs (~19 kbp) from DNA isolated from phage-like particles in the Poulsonii clade strains *s*Mel and its close relative *s*Neb (a.k.a. ‘NSRO’; original host=*Drosophila nebulosa*). Regions with substantial homology to the *s*Mel phage-like contig are detected in most *s*Mel assemblies [123], suggesting that they might be lysogenic. We searched for the presence of such phage-like contigs in the *s*Ald-Tx, *s*Hy2 and *s*Moj genome assemblies by Geneious ‘Map to reference’ and blastn of assembled contigs to the *s*Neb and *s*Mel phage-like sequences. The only result from this search was RXFZ02000001 (23,369 bp) in *s*Hy2, which is also the only contig effectively contributed by the long-read (MinION) dataset (Table 1). RXFZ02000001 contained several genes associated with phage genomes, such as portal, head-tail connector, capsid protein, recT, terminase (large and small subunits) and transposase-like proteins (Fig. S5) and had higher coverage (approximately ten times higher than regions of contigs that are not repetitive, based on the short-read dataset; not shown). Nucleotide similarity between *s*Hy2’s RXFZ02000001 and the *s*Mel and *s*Neb phage-like contigs ranges from ~65 to 72% (Fig. S5; alignment available in Dataset S8).

#### Annotations based on COG and KEGG

Based on the COG analysis of the three *Drosophila*-associated Citri clade strains, the most abundant category of genes was ‘Translation, ribosomal structure and biogenesis’ (COG=J), followed by ‘Replication, recombination and repair’ (COG=L) (Fig. S6). This pattern was reversed (i.e. category L had the most genes, followed by J) in the Poulsonii clade strains (*s*Mel, *s*Hy1 and *s*Neo), but it appears to be driven by the large number of genes annotated as transposases, which fall under L (Dataset S9).

Respectively for *s*Ald-Tx, *s*Moj and *s*Hy2, of the predicted protein-coding genes, 432, 423 and 416 could be assigned functional predictions in the form of KO numbers. A Venn diagram comparison of the genes assigned KO numbers indicates that the three strains share 360 genes (Fig. S7 and Dataset S10); these analyses only count one copy of each KO number per strain. *s*Ald-Tx and *s*Moj, which are each other’s closest sister (Fig. 2) but whose hosts (*D. aldrichi*, *D. mulleri* and *D. mojavensis*) are members of the *mulleri* complex, share the most KO genes (360+17; Fig. S7). The number of strain-unique KO genes ranged from 9 to 12. Based on BRITE categories, the largest differences in KO gene content amongst the three strains are in the category of enzymes (Fig. S8 and Dataset S11). For comparison, the three Poulsonii clade strains *s*Mel, *s*Hy1 and *s*Neo, respectively, had 635, 553 and 520 genes assigned KO numbers. Of these, 413 are shared, and four to nine are strain-unique (Fig. S9 and Dataset S12).

Below, we compare particular sets of genes amongst five strains of the Citri clade and five strains of the Poulsonii clade. For the Citri clade, we included *s*Nigra (host: non-*Drosophila* dipteran), the three *Drosophila*-associated strains (*s*Ald-Tx, *s*Hy2 and *s*Moj), and *S. melliferum* (a culturable and horizontally transmitted symbiont of the honey bee that has comparatively greater metabolic capacities). For the Poulsonii clade, we include the reference genome for each of the three available *Drosophila*-associated strains (*s*Mel, *s*Hy1 and *s*Neo), their sister lineage *s*Car (from the flesh fly *S. carnaria*) and a more distant relative (*s*Fus) associated with the tsetse fly (*G. fuscipes fuscipes*).

#### DNA repair-related genes

Within the Homologous Recombination and the Base Excision Repair pathways, the *Drosophila*-associated Citri clade strains tend to have more missing genes than their Poulsonii clade counterparts (Table S7). Previous research on the Citri clade strains *S. melliferum* and *S. citri* indicates that they are RecA-deficient, rendering them highly sensitive to UV radiation [125]. *RecA* was frame-shifted, incomplete or absent in the five Citri clade genomes compared (Table S7) and appears to be lacking in all the Citri clade assemblies (not shown). In contrast, all Poulsonii clades except *s*Mel [126] and sCru appear to have a functional copy of *RecA* (not shown). Little to no difference in gene content is detected amongst the ten strains compared regarding the Nucleotide Excision Repair and the Mismatch Repair pathways. A notable difference between the two clades is that the five Citri clade strains compared in Table S7 encode deoxyribodipyrimidine photo-lyase (*phrB*), whereas all five Poulsonii clade strains do not. Nonetheless, *phrB* is encoded by Poulsonii clade strains TU-14 and *S*. sp. NBRC_100390 (not shown), the closest known relatives of *s*Fus (Fig. 2), suggesting independent losses in *s*Fus and in the ancestor of *Poulsoni* s.s. In general terms, it appears that the *Drosophila*-associated Citri clade strains have similar or worse abilities to repair DNA and are thus likely to evolve equally or more rapidly than their Poulsonii clade counterparts, which have amongst the highest DNA substitution rates of bacteria [39].

#### Inferred abilities to import and process certain metabolites

The ability to import and process molecules associated with energy metabolism appears to be substantially limited in the *Drosophila*-associated Citri clade strains (*s*Ald-Tx, *s*Hy2 and *s*Moj; summarized in Fig. S10). As detailed below, the annotation suggests that the only sugar that they are able to import (and metabolize) is fructose. Comparatively, at least one of the three members of the *Drosophila*-associated Poulsonii clade is predicted to be able to convert pyruvate to acetyl-CoA (pyruvate oxidation; Fig. 2) and to import and process glucose (functionally confirmed in *s*Mel [126]), fructose (except *s*Mel), glycerol (which is predicted to produce peroxide) and arginine (only *s*Hy1).

Of the ten strains compared in Table S8, the Citri clade *S. melliferum* and *s*Nigra and the Poulsonii clade *s*Hy1, *s*Mel, *s*Neo and *s*Fus are predicted to be able to import and process glucose (i.e. they have putatively functional *ptsG*, *crr* and *pgi* genes). In contrast, the remnants of at least two of these three genes appear non-functional in the *Drosophila*-associated Citri clade strains (*s*Ald-Tx, *s*Hy2 and *s*Moj) and in the Poulsonii clade *s*Car, suggesting that they cannot use glucose as an energy source, unless they can import glucose employing other putative sugar transporters encoded by their genomes (e.g. locus tags EHU54_02420, EHU54_02680, EHV01_02065, EHV01_02360, EHV01_01190, BST80_02715 and BST80_01055). All Citri and Poulsonii strains compared, except for *s*Mel, are predicted to be able to import and metabolize fructose, with all strains except *s*Mel, *s*Hy1 and *s*Neo encoding two putatively functional *fruA* genes (Fig. S10 and Table S8); *s*Neo and *s*Hy1 retain one functional *fruA* and non-functional remnants of another putative *fruA* gene. Of the ten strains compared, only *s*Hy2 (EHV01_02360) and *S. melliferum* have a putatively functional importer of trehalose (*treB*), but all strains compared except *S. melliferum* lack a functional *treA* gene (the few other *Spiroplasma* strains that appear to encode *treA* and/or *treB* are listed in Dataset S13). Therefore, none of the Diptera-associated strains that we compared in Table S8 seem to be able to use trehalose, the main sugar in insect haemolymph (reviewed in [127]). Of the ten strains compared, only *S. melliferum* encodes all four genes needed for uptake and metabolism of *N*-acetylmuramic acid (i.e. *murP*, *murQ*, *nagA* and n*agB*). The ability to import and metabolize glycerol or produce H_2_O_2_ (based on the presence of genes encoding *glpF*, *glpK* and *glpO*) appears to be restricted to *S. melliferum* (Citri clade), and all but one (*s*Fus) of the five Poulsonii clade strains that were compared.

Of the ten strains compared, only *s*Hy1 and *S. melliferum* appear to encode the machinery needed to import and metabolize arginine (i.e. the adjacent genes *ArcA*, *ArcF*, *ArcC* and *ArcD*) (Fig. S10 and Table S8). Of the ten strains compared, all but the *Drosophila*-associated Citri clade (*s*Ald-Tx, *s*Hy2 and *s*Moj) encode the full operon associated with pyruvate oxidation (pyruvate=>acetyl CoA; Koala M00307; *pdhA* is pseudogenized, *pdhB* and *pdhD* are absent and *pdhC* is complete) (Table S8 and Fig. 2). All the strains compared, except *S. melliferum*, lack functional copies of at least one of the five genes needed to import/process cellobiose (Fig. S10 and Table S8). *GlpX* (K02446, which catalyses GA-P3>>Fructose-6-P) appears to be missing from the five Poulsonii strains compared (Table S8), but it is present in NBRC_100390 and TU-14 (WP_070407118.1). Of the five Citri clade strains compared, *GlpX* is present in *S. melliferum*, *s*Nigra and *s*Moj, but it is pseudogenized in *s*Ald-Tx and *s*Hy2 (Table S8 and Fig. 2).

The *Drosophila*-associated Citri clade strains (*s*Ald-Tx, *s*Hy2 and *s*Moj) appear to be more limited than their Poulsonii clade counterparts (*s*Mel and *s*Hy1) regarding the ability to generate phospholipids from fatty acids or diacylglycerol. Of the seven genes involved in the nonmevalonate terpenoid synthesis pathway, the ten strains compared encode them all, except for *s*Ald-Tx and *s*Hy2, in which *ispH* (K03527) is pseudogenized (Table S8). The KEGG database indicates that all *Spiroplasma* lack the enzyme that converts phosphatidyl-glycerophosphate to phosphatidyl-glycerol (EC 3.1.3.27), which is one of the steps in the DAC-3P to cardiolipin pathway (see KEGG map00564). However, Paredes *et al*. [126] propose that *s*Mel’s WP_258267084.1 (annotated as a lysophospholipase) catalyses this reaction. Orthologues of WP_258267084.1 are found in several other Poulsonii and Citri clade strains (e.g. *s*Hy1 and *S. melliferum*, respectively) but are pseudogenized in *s*Ald-Tx, *s*Hy2 and *s*Moj (Table S8), implying that they are unable to synthesize cardiolipin from DAC-3P. Three additional genes involved in glycerophospholipid and/or glycerolipid metabolism (i.e. *plsC*, *plsY* and *plsX*) appear to be complete in the ten strains compared (Table S8).

The three *Drosophila*-associated Citri clade strains lack a functional copy of ferritin-like genes (*Ftn*), which are present in the remaining seven strains compared in Table S8. Ferritin-like proteins are involved in iron sequestration [128]. A search for the term ‘ferritin’ in the gene annotations of the recently released genome assemblies of the Citri and Poulsonii clades indicates three additional strains lacking this gene, which appear to have independently lost it (i.e. *s*Pub, *s*Cru and *s*Ten; not shown). Two observations suggest that an *Ftn* gene is important for the *sMel-D. melanogaster* symbiosis: *Ftn* transcript levels of *s*Mel are higher when it is inside the host compared to when it is cultivated outside of the host [23], and *Ftn* is one of few genes whose protein abundance is upwardly biassed compared to transcript levels [42]. Iron homeostasis is relevant to insect-symbiont associations (e.g. [129]), including that of *Drosophila* and *Spiroplasma*. Strain *s*Mel induces expression of *Drosophila* transferrin gene *Tsf1*, which binds and facilitates the sequestration of iron from the haemolymph to the fat body [35,130]. The proliferation of *Spiroplasma* in *D. melanogaster* (both *s*Mel and the plant pathogen *S. citri*) requires Tsf1-bound iron [130]. The lower levels of free iron in the haemolymph appear to underlie the *s*Mel-induced resistance of *D. melanogaster* against two pathogens (the bacterium *Providencia alcalifaciens* and the fungus *Rhizopus oryzae* [35]). If the *Spiroplasma*-encoded *Ftn* genes are involved in the ability of *Spiroplasma* to exploit iron, the *Drosophila*-associated Citri clade strains (*s*Ald-Tx, *s*Hy2 and *s*Moj) likely have limited iron exploitation capacities.

Therefore, overall, the *Drosophila*-associated Citri clade strains (*s*Ald-Tx, *s*Hy2 and *s*Moj) seem metabolically more limited than their Poulsonii clade counterparts (*s*Mel, *s*Hy1 and *s*Neo) regarding the importation and processing of several metabolites including sugars, lipids and iron. The inability to exploit such resources may underlie their comparatively lower densities and vertical transmission rates in native and non-native hosts [38,102, 131 and personal observation].

#### Putative virulence factors

The genomes of *Spiroplasma* encode a diverse set of known or putative toxin genes, some of which have been mechanistically linked to phenotypes, such as male killing and parasite killing [19,43, 46, 47, 51, 77, 97, 112, 114, 126, 132,135]. Amongst these, genes with domains annotated as RIPs appear to be the most common and diverse and are predominantly found in vertically transmitted *Spiroplasma*, based on a comparison of 12 vertically and 31 horizontally transmitted *Spiroplasma* genomes available at that time [19]. RIPs include toxins such as ricin (from the castor oil plant) and Shiga toxin (from *E. coli*). RIPs target an adenine found within a 12-nucleotide motif of the 28S rRNA that is universally conserved in eukaryotes, termed the sarcin-ricin loop. RIPs remove the target adenine, leaving an abasic (a.k.a. depurinated) site, which irreversibly renders the ribosome non-functional and thus stalls protein synthesis (reviewed in [136]). The toxicity of RIPs varies based on their ability to enter the cell, to reach the appropriate cellular compartment and to resist degradation [137]. Evidence that the presence of *Spiroplasma* induces ribosome depurination exists for two *Drosophila*-associated Poulsonii clade strains. The presence of *s*Neo in its *Drosophila* host confers protection against nematodes and wasps; both macroparasites exhibit signals of ribosome depurination in the presence of *s*Neo, and a recombinantly produced RIP protein encoded by sNeo (i.e. *s*Neo_RIP1_WP_127093322; Fig. S11 and Dataset S14) has confirmed RIP activity, as it depurinates the target adenine *in vitro* of both whole nematode and cell-free rabbit ribosomes [43,97]. Similarly, the presence of *s*Mel in its *Drosophila* host confers protection against certain wasps; such susceptible wasps exhibit signs of ribosome depurination, depurination of *Drosophila* ribosomes is also detected (at least at the embryo stage) and ectopic expression of two *s*Mel-encoded RIP genes (i.e. MSRO_RIP1_WP_040093770 and MSRO_RIP2_WP_040093936; Fig. S11) confirms their RIP activity against host ribosomes [51,91, 92, 96, 97, 133, 134].

Our results reveal the presence of domains identified as RIPs in numerous additional *Spiroplasma* strains, including the three *Drosophila*-associated Citri clade strains: *s*Moj (one gene), *s*Ald-Tx (two genes) and *s*Hy2 (four genes) (Figs 2 and S11). All but five Citri clade strains encode at least one RIP gene, including several first records (*s*Pte, *s*Nob, *s*Chi, *s*Pub and *s*Tum). All but one Poulsonii clade strain (i.e. *s*Dum) have at least one RIP gene, including several first records (*s*Car, TU-14, NBRC_100390, *s*Fus, *s*Cru, *s*Ten and *s*Cal). Similarly, in the newly identified Clade X, all but one strain had at least one RIP gene. Several new RIP gene records were found in the Ixodetis and Apis clades (Fig. S11).

Although we recovered 175 RIP domain sequences, 31 sequences were excluded from phylogenetic analyses because they were very short (Dataset S14). Many nodes in the RIP domain amino acid phylogeny received low support by one or more of the clade support measures (Fig. S11). Whilst there are a few RIP domain clades that are restricted to the same *Spiroplasma* strain or *Spiroplasma* clade, there are several cases where RIP domains recovered as sister lineages belong to distantly related *Spiroplasma* strains. For example, the single *s*Moj RIP gene (*s*Moj_RIP1_MBH8624287) appears closely related to RIPs from Clade X (*s*Lun), Citri (*s*Had) and Poulsonii (*s*Hy1 WP_198049692 and *s*Mel WP_040093770). One RIP from *s*Hy2 (*s*Hy2_EHV01_04225) was recovered as sister to a RIP from Clade X (*s*Cur_WP_342223867) and was closely related to RIPs from other Clade X (sLute_WP_338968587 and sLun_WP_338981923) and from Poulsonii (sNeo_RIP4_WP_158676203). One RIP from *s*Ald-Tx (*s*Ald_Tx_EHU54_01310) appears as a sister to the RIP of *s*Cerv (Citri), with their next most closely related RIPs belonging to Clade X (*s*Flav, *s*Cur and *s*Lute).

**Fig. 3.**
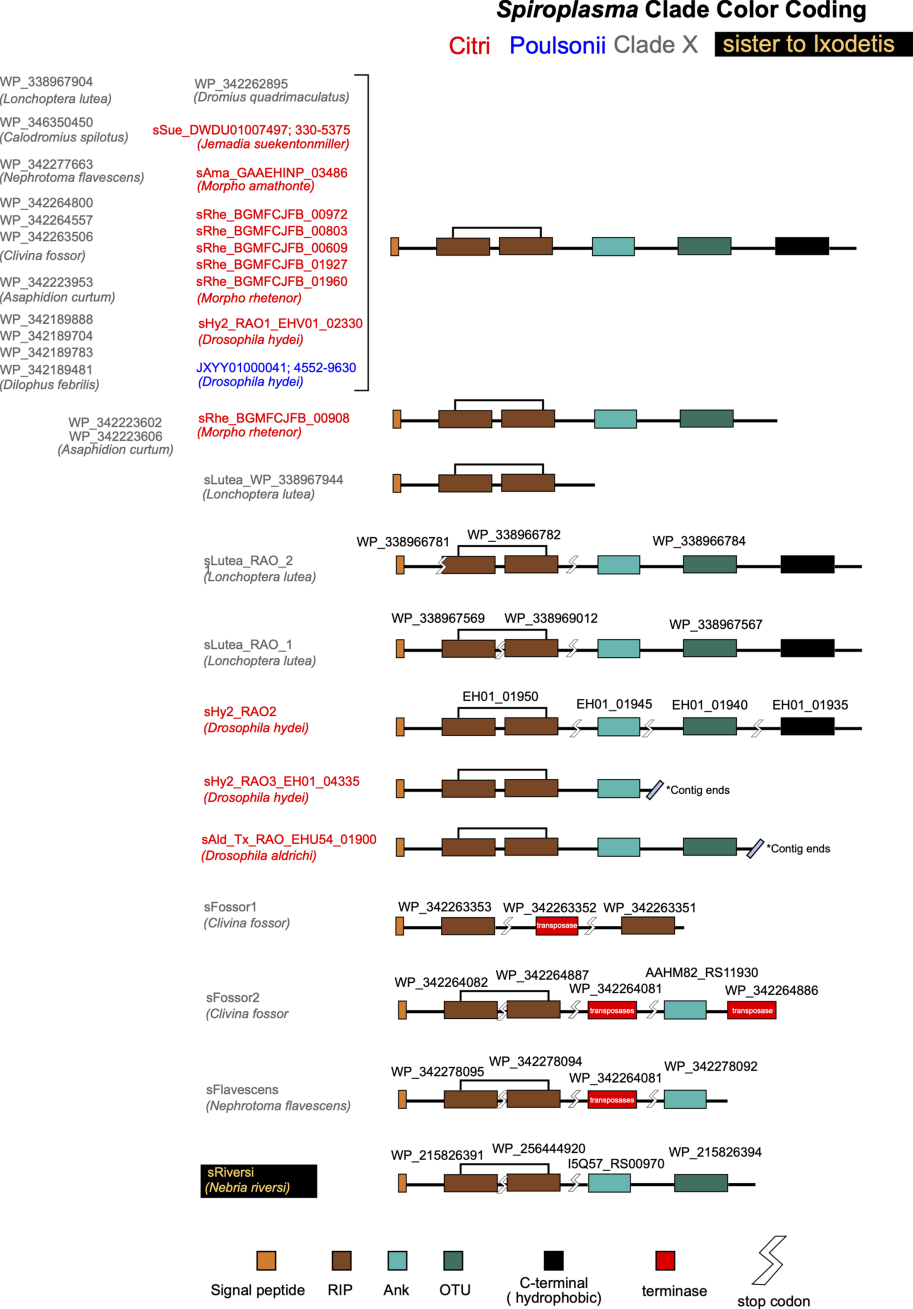
Domain architecture (not to scale) of genes in the clade RAO (RIP, ankyrin and OTU).

The remaining RIP-encoding genes from *s*Hy2 (three genes) and *s*Ald-Tx (one gene) fell within a well-supported clade (‘Clade RAO’; Fig. S11). Most of the genes in this clade have an unusual domain structure, previously referred to as ‘Spaid-like’ by Moore and Ballinger [19] and ‘RIP/Spaid’ by Filée *et al*. [114], containing two RIP domains, followed by ankyrin repeats (A), an OTU domain (ovarian tumour deubiquitinase; IPR003323; O) and a C-terminal hydrophobic (transmembrane) domain (Fig. 3). Clade RAO is composed predominantly of genes from clades X (first record) and Citri, as well as one strain from each of three additional *Spiroplasma* clades: Ixodetis (*s*Chrys WP_174481319; not shown), ‘sister to Ixodetis’ [the *s*Riversi WP_215825920 (Fig. S11) and WP_215826391; not shown] and Poulsonii. The Poulsonii clade RAO gene (*s*Hy1_RIP2_MBH8623170) appears most closely related to *s*Hy2_EHV01_02330 (Citri), suggesting a horizontal transfer between these distantly related strains that overlap geographically and share the same host species [27,30, 31, 52]. Another *s*Hy2 RIP (*s*Hy2_EHV01_01950) is recovered as closely related to RIPs from several Citri clade strains associated with Lepidoptera, and Clade X strains associated with Diptera and Coleoptera.

To further explore potential patterns of horizontal gene transfer, we examined the DNA sequences of clade RAO genes, including adjacent regions in cases where a stop codon or transposase gene interrupts domains. We arbitrarily broke up the alignment into five blocks that were separated by several poorly conserved regions (based on visual examination of the identity graph in Geneious; Dataset S15). We performed phylogenetic analyses on each of these five regions, which revealed patterns of topological incongruence amongst regions (Fig. S12). We highlight, for example, that in the first block (N-terminus), *s*Hy2_EHV01_02330 appears as most closely related to RAO in *s*Hy1, consistent with the amino acid phylogeny of only the RIP domain (Fig. S11). In contrast, the ankyrin repeat region of *s*Hy2_EHV01_02330 appears most closely related to two Clade X RAOs from strain sFossor (WP_342264303 and WP_34226455). These observations are consistent with the features of polymorphic toxins, i.e. multidomain secreted proteins that diversify through recombination and domain swapping, and tend to be associated with horizontally transferred elements [77,138].

OTU domains, which are common in eukaryotic genes, with and without RIP and/or ankyrin domains have previously been reported in genes of several *Spiroplasma* strains (e.g. [19,77, 112, 114, 126, 132]), including in *Spaid* (ankyrin+OTU male-killing gene of *s*Mel [47]). According to the analysis of Moore and Ballinger [19], within *Spiroplasma*, OTU and ankyrin repeat domains are exclusive to vertically transmitted strains. In addition to OTU domains present in ‘RAO’ genes, we detected OTU domains in several additional Citri clade strains (e.g. *s*Moj, *s*Tum and *s*Man) and Clade X (Fig. 2). Within the Poulsonii clade, however, OTU domains are only detected in the three *Drosophila*-associated strains (Fig. 2), which are known to be vertically transmitted. Within the Citri clade, it is notable that OTU domains are absent in the plant associates (e.g. *S. phoeniceum*, *S. kunkelii*, *S. citri* and *S.* sp. Chis) and the horizontally transmitted pathogen of honeybees (*S. melliferum*). All other Citri clade strains have at least one OTU domain-containing gene and are associated with insects (Diptera, Lepidoptera, Hymenoptera and one Coleoptera; Fig. 2). The transmission mode of most of these newly available strains is unknown (most were discovered in the process of assembling and annotating their host’s genome sequence), but given the presence of OTU and ankyrin repeat domains, they are likely vertically transmitted. The OTU domain tree recovers a clade comprised exclusively of all but four of the OTU-containing genes from *Drosophila*-associated strains (both Poulsonii and Citri clades; Fig. S13 and Dataset S16), including *Spaid* (WP_105629072), which could indicate a common origin or function. The exceptions are *s*Hy1dLiv_PMBNAAIA_00948_AO (sister to an OTU from a Citri clade *Myrmica* ant associate), *s*Hy2_EHV01_02330_RAO (sister to Citri clade strains associated with Lepidoptera), *s*Hy2_EHV01_01940 (sister to a Citri clade strain associated with a *Morpho* butterfly) and sAld_EHU54_01900_RAO (sister, albeit with low support, to two genes from a Clade X strain, and embedded within a well-supported clade that includes Citri clade strains from Lepidoptera and Coleoptera). In contrast, the RIP domain of *s*Ald_EHU54_01900_RAO is closely related to genes from *s*Hy1 (Poulsonii), *s*Hy2 and *s*Teh (Citri clade) (Fig. S11), suggesting that *s*Ald_EHU54_01900 is the result of horizontal transfer and domain shuffling.

Strains *s*Ald-Tx and *s*Hy2 encode a gene containing an OTU domain and another toxin domain (i.e. ETX/MTX2; *β*-pore-forming toxins with receptor-binding activity; hereafter ‘ETX’), an unusual domain architecture that was not found in any other *Spiroplasma* except *s*Hy1 (sHy1_00057 [39]). Genes encoding components of the ETX pore-forming protein have been reported in several *Spiroplasma* genomes (reviewed in [77]) and tend to be associated with vertically transmitted strains [19]. We detected the ETX domain in additional strains from the following clades: several from Poulsonii, all but two strains of Citri (only absent in the plant associates *S. phoeniceum* and *S. kunkelii*), Mirum, all but one of Clade X strains (Fig. 2), Apis and Ixodetis (Fig. S14). The ETX domain of *s*Ald-Tx, *s*Hy2 and the Poulsonii-clade *s*Hy1 was highly similar, including adjacent regions (Fig. S15 and Dataset S18), and grouped together (Fig. S14 and Dataset S17), implying a recent horizontal transfer between the Poulsonii and Citri clade. These were, in turn, sister to a clade composed of genes in other strains of the Citri clade, as well as strains in Clade X, and one strain ‘sister to ixodetis’, collectively associated with Diptera, Coleoptera and Hymenoptera. The ETX domain-containing gene of *s*Moj (BST80_00145=MBH8623564.1) was most similar to that of a Clade X strain associated with a beetle (WP_342264206.1). With the exception of ETX genes in the Mirum clade, there is a general lack of monophyly of ETX domains within *Spiroplasma* strains and clades, suggestive of substantial losses and gains from distantly related strains.

A gene encoding an ETX domain, along with an upstream PQQ-like domain (PFAM: PF13570) and an N-terminal signal peptide, is found in the Poulsonii clade strain *s*Neo (WP_127092276) and the Citri clade strain *s*Nigra (WP_126821430), both from dipteran hosts associated with mushrooms. We found evidence of this PQQ-ETX domain architecture in one Clade X strain associated with a beetle, and in six additional Citri clade strains (*s*Chi, *s*Rhe, *s*Ach, *s*Ama, *s*Tum and *s*Man), but not in those associated with *Drosophila* (Fig. 2). Additional strains encoding a PQQ domain-containing gene, but lacking an ETX domain, were detected in clades Poulsonii, Citri, Chrysopicola, Mirum and X (Fig. 2). In *s*Mel (Poulsonii), the neighbouring CDS WP_040094248.1 (PQQ) and WP_040094250.1 (ETX) appear to reflect the remnant of a PQQ-ETX protein. A phylogeny of the PQQ domain alignment (Fig. S16 and Dataset S19) had very poor resolution and is thus not shown. The function of PQQ-domain-containing proteins in *Spiroplasma* is unknown. PQQ-like enzymes have repeats of a ß propeller. Depending on the number of blades that they contain, ß propellers have a diversity of functions, including ligand binding, transferase, hydrolase, lyase, isomerase, signalling, oxidoreductase and structural protein [139].

The widespread occurrence in *Spiroplasma* of genes with the RAO domain architecture, whose RIP domain region forms a monophyletic group, implying a common origin, is intriguing. Ankyrin repeats are a common structural motif (33 aa) involved in protein-protein interactions. They are ubiquitous in eukaryotic proteins but also found in viruses and archaea, and they are particularly common in bacteria with a symbiotic (pathogenic to mutualistic) lifestyle [140]. In *Spaid*, the male-killing gene of strain *s*Mel, the ankyrin repeat domain is required for Spaid to accumulate on the male X chromosome, a prerequisite for its male-killing action [47]. Similarly, in the human pathogen *Legionella pneumophila*, the ankyrin repeat domain of a particular protein is used to specifically bind to a host protein on which damage (i.e. phosphocholination) is exerted by another domain of the same protein [141]. Therefore, we hypothesize that through its protein-binding function, the ankyrin repeat domain of RAO and other *Spiroplasma* genes enables localization to specific eukaryotic cells or subcellular regions. Because of the apparent role of at least some *Spiroplasma* RIPs in host defence, ankyrin repeats might enable the selective localization of RIP (whose ribosomal RNA target motif is universally conserved in eukaryotes) and its depurination activity, to the cell or cell compartments of the endo-macroparasite (e.g. parasitic wasp or nematode), rather than to those of the host. Regarding the potential role of OTU in RAO, the recent work on *Spaid* by Harumoto [46] offers potential clues. OTUs encode deubiquitinase activity, which reverses protein ubiquitination, an important post-translational modification in eukaryotes, that influences stability, interactions, activity and localization of proteins. Harumoto [46] demonstrated the following: (1) *Spaid* lacking the OTU domain exhibits an attenuation of the male-killing action, because the OTU-free Spaid protein is polyubiquitinated and degraded via the host ubiquitin-proteasome pathway, and (2) Spaid uses its OTU domain to deubiquitinate itself in an intermolecular fashion, presumably through a homomeric interaction. Consequently, the OTU domain functions as a self-stabilizing mechanism for Spaid. We hypothesize that the OTU domain in RAO and other *Spiroplasma* genes could serve such a self-stabilizing role.

Our results indicate that the genomes of the *Drosophila*-associated Citri clade strains *s*Moj, *s*Ald-Tx and *s*Hy2 encode a putative toxin that, within *Mollicutes*, appears to be exclusive to the Citri clade: ADP-ribosyltransferase exoenzyme, PFAM:PF03496 or lethal factor (Fig. S17 and Dataset S20). ADPs convert NAD into nicotinamide and ADP ribose, which is transferred to proteins, nucleic acids or small molecules, and are found in all kingdoms of life (reviewed in [142]). Such modifications can inhibit the normal function of host proteins or activate host proteins in a manner that promotes bacterial fitness [143,146]. ADP is typically part of a binary toxin that also includes the PA protein that enables entry into the host’s cell (reviewed in [143]). Of all *Spiroplasma* sequenced to date, only *S. melliferum* encodes putative functional genes for both components (Fig. S17), but it was not found to cause the expected cytotoxic effect in the yeast growth-deficiency assay, suggesting that it requires a host-specific factor for activation [144]. Ten additional Citri clade species/strains encode remnants of one or both genes (Fig. S17), all of which occur immediately downstream of two apparently functional CDSs (not shown): an NAD(+)/NADH kinase (GO function: 0003951; GO_process: GO:0006741) and a PTS transporter subunit EIIB (GO function: 0008982). The three *Drosophila*-associated Citri clade strains encode a full-length ADP (annotated as ‘lethal factor CDS’ in Fig. S17). However, the PA gene is interrupted by an early stop codon (only retaining one of the three domains; the Ca-binding domain pfam03495). Immediately downstream of the stop codon, *s*Moj has a remnant of a transposase (BST80_01070; PF13613), whereas in *s*Ald-Tx and *s*Hy2, the end of the contig is reached, likely reflecting the presence of a repetitive sequence that hampered assembly of this region. Remnants of PA’s domain 2 (pfam17475) and domain 3 (pfam17476) are found in some lineages (Fig. S17). The *Spiroplasma* ADP proteins resemble the C3-like ADP-ribosyltransferases, as evidenced by the conservation of two residue sites essential for enzymatic activity: the R site and the QXE motif (Fig. S17). No evidence of signal or transport motifs exists in any of the *Spiroplasma* ADP-domain-containing genes, raising doubts about their potential function, unless they function within the *Spiroplasma* cell. All of the *Spiroplasma* ADP genes share high homology with each other and are very distinct from their closest blast hits (i.e. from *Bacillus*; not shown).

Amongst *Spiroplasma* proteins considered important for the interaction with the (insect) host are the abundant membrane lectins referred to as spiralins [147,149]. Each of the three *Drosophila*-associated Citri clade strains (*s*Ald-Tx, *s*Moj and *s*Hy2) encodes one gene annotated as the lipoprotein spiralin (hereafter *spiA*; Table S8). These spiralin gene sequences formed a monophyletic group, whose closest relatives are spiralin genes from other members of the Citri clade (Fig. S18 spiralin and Dataset S21), including a gene known to be required for efficient transmission by the insect vector to the host plant of *S. citri* [148]. In contrast, the six Poulsonii clade strains, including *s*Fus from *Glossina*, encode at least two highly divergent spiralin genes (Fig. S18), identified in *s*Mel as *spiB* [126] and *spiC* [23]. In addition, all Poulsonii clade strains except *s*Fus encode a spiralin gene that is more similar to those of the Citri clade (i.e. *spiA*). The *spiB* gene is the most highly expressed gene in *s*Mel [23,126], and it appears to be involved in the process of vertical transmission during which *s*Mel enters the oocyte from the haemolymph [149]. It is possible that lacking a *spiB* or *spiC* homologue contributes to the lower vertical transmission efficiency of the *Drosophila*-associated Citri clade strains.

## Conclusions

Based on the draft assemblies contributed here, we infer that compared to their Poulsonii clade counterparts (*s*Mel, *s*Hy1 and *s*Neo), the *Drosophila*-associated Citri clade strains (*s*Ald-Tx, *s*Hy2 and *s*Moj) have more limited metabolic capacities (including importation and metabolism of sugars, lipids and iron), possibly lower ability to transmit vertically due to the absence of the *spiB* gene and equally bad or worse DNA repair mechanisms. Collectively, the above features may underlie their comparatively lower densities and vertical transmission rates and lead us to predict that the *Drosophila*-associated Citri clade evolves at rates similarly high to those reported in the Poulsonii clade. Frequent loss and rapid evolution of symbionts, along with the lack of *in vitro* culture protocols and genetic tractability, pose practical challenges to experiments assessing phenotypes and their underlying genetic basis. Notwithstanding their comparatively ‘poor’ features, the genomes of *s*Ald-Tx, *s*Hy2 and *s*Moj collectively encode a similarly diverse repertoire of putative toxin genes to the Poulsonii clade, with evidence of substantial exchanges, including between the Poulsonii and Citri clade (e.g. the RAO gene of *s*Hy1 and *s*Hy2), and within-genome shuffling. Presumably, some of these toxins are used in the interaction with their *Drosophila* host or their host’s natural enemies, but based on examination of two strains (*s*Ald-Tx and *s*Moj) in the presence/absence of two different parasitic wasps (including one highly susceptible to Poulsonii clade strains), no evidence of fitness consequences to their native hosts has been detected. How Citri clade strains persist, and even achieve high prevalence, in (certain) *Drosophila* populations remains a mystery. It is possible that they confer yet undiscovered net fitness benefits to their (female) hosts or that they rely on substantial horizontal transmission. Beyond the *Drosophila*-associated Citri clade, the discovery of a divergent *Spiroplasma* lineage associated with dipterans and coleopterans (Clade X) underscores the cryptic diversity of endosymbionts that metazoan genome projects are uncovering. The common occurrence of RAO domain architecture in very distant *Spiroplasma* lineages (predominantly in clades X and Citri) suggests that it plays an important role in insect-*Spiroplasma* interactions, which may include a combination of ribosomal RNA depurination (by RIP), selective localization to target cells or cell compartments (by ankyrin repeat proteins) and self-stabilization (by the OTU deubiquitinase).

## Supplementary material

10.1099/mgen.0.001408 Table S1.

10.1099/mgen.0.001408 Uncited Table S2.

## Abbreviations

BUSCO: Benchmarking Universal Single-Copy Orthologs
COG: Cluster of Orthologous Groups
COI: cytochrome oxidase I
KEGG: Kyoto Encyclopedia of Genes and Genomes
KO: KEGG Orthology
NCBI: National Center for Biotechnology Information
OTU: ovarian tumour deubiquitinase
PA: protective antigen
PGAP: Prokaryotic Genome Annotation Pipeline
RIPs: ribosome-inactivating proteins
WGS: whole-genome shotgun
