# Supplementary material for: Diverse toxin repertoire but limited metabolic capacities inferred from the draft genome assemblies of three Spiroplasma (Citri clade) strains associated with Drosophila
Source: Microb Genom. 2025 Jun 5;11(6):001408. doi: 10.1099/mgen.0.001408 (PMC12453411; doi:10.1099/mgen.0.001408)
Supplement: Table S1. [file mgen-11-01408-s001.pdf]

Table S1. Insect species and strains/isofemale lines used for genome assembly and/or fitness assays.

**Insect Species**	**Strain or isofemale line (year established)**	***Spiroplasma* strain**	**Location captured**
Drosophila aldrichi	Fr0317-09 (2017)	*Spiroplasma* sAld-Tx	San Marcos, Texas
Drosophila hydei	H25 (2014–2015)	*Spiroplasma* sHy2	Mexico
Drosophila mojavensis	CI-33-15 (2012)	*Spiroplasma* sMoj	Catalina Island, California
Leptopilina heterotoma	Lh14 (2002)	none	Winters, California
Asobara sp.	w35 (2012)	none	San Marcos, Texas

Table S2. Restriction enzymes and conditions to differentiate *Spiroplasma* strains *s*Hy1 (*Poulsonii* clade) and *s*Hy2 (*Citri* clade) at the 16S rRNA gene.

Restriction Enzyme	Cut site	Temp (°C)	*s*Hy1 fragment sizes (bp)	*s*Hy2 fragment sizes (bp)
*Bsa*I	5'...GGT CTC (1/5)^...3'	37	1,216<br>161	No cut
*Sac*I	5'...GAG CT/C...3'	37	No cut	818<br>550

Supporting Table S5. ***Spiroplasma*** **frequencies in wild-caught** ***D. aldrichi*** **and** ***D. mulleri*** **from sampled localities of Texas.**

**Locality (Texas, USA)**	**Latitude**	**Longitude**	**Dates**	**Species**	***Spiroplasma*-positive**	***Spiroplasma*-negative**	**Total**	**Frequency**
Brackenridge Field Lab, Austin	30.284751	-97.778115	10–12 November 2013	*D. aldrichi*	31	2	33	0.94
				D. mulleri	24	8	32	0.75
Freeman Ranch, San Marcos	29.935278	-98.014284	March–May 2012	*D. aldrichi*	13	10	23	0.57
				D. mulleri	7	13	20	0.35
			14–18 March 2016	*D. aldrichi*	14	7	21	0.67
				D. mulleri	1	25	26	0.04
San Antonio	29.676634	-98.475748	12–18 March 2012	*D. aldrichi*	1	5	6	0.17
				D. mulleri	1	8	9	0.11
**Total for *D. aldrichi***	**all**	**all**	**all**	***D. aldrichi***	**59**	**24**	**83**	**0.71**
**Total for *D. mulleri***	**all**	**all**	**all**	***D. mulleri***	**33**	**54**	**87**	**0.38**

Supporting Table S6. P-values and assumed distribution (binomial or quasibinomial) from Analysis of Deviance Table (Type II tests) for the experiments that tested whether *Spiroplasma s*Moj influenced the interaction between *Drosophila mojavensis* and two species of parasitic wasps. None of the p-values were significant after Bonferroni correction for multiple comparisons (adjusted alfa = 0.05/11 = 0.0045).

**Wasp treatment**	**larva to adult fly survival**		**larva to pupa survival**		**wasp success (adult wasp over fly larvae)**		**pupal failure (failed pupae over pupae)**	
no wasp	0.9554	quasibinomial	0.3434	quasibinomial	n/a	n/a	0.5153	quasibinomial
Leptopilina_Lh14	0.9949	binomial	0.1191	quasibinomial	0.5552	quasibinomial	0.7988	quasibinomial
Asobara_w35	0.1199	quasibinomial	0.04018	quasibinomial	0.504	quasibinomial	0.7941	quasibinomial

CTAB Extraction Protocol

1. Place CTAB buffer in 60°C water bath for 30 min before use
2. Place tissue or specimen in 1.5-ml microtube
3. Add 300ul CTAB buffer to each tube
4. Grind specimen with pestle.
5. Cover tubes and incubate at 60°C for at least 1h
6. Add 300ul of sterile molecular grade water
7. Add 300ul of phenol, mix well, centrifuge for 10 min. Extract supernatant (top layer) and place in a new tube.
8. Add 300ul phenol-chloroform-isoamyl, mix well, and centrifuge for 10 min. Extract supernatant (top layer) and place in a new tube.
9. Add 300ul chloroform-isoamyl (24:1) to each tube, and shake by inverting for 2 min. Centrifuge for 10 min.
10. Label a new set of 1.5ml microtubes. Add 25ul 3M NaOAc and 600ul of 100% Ethanol (kept at -80C freezer) to each tube
11. Take aqueous (top) phase from step 9 and add to the tubes from step 10. Be sure not to disturb the interphase to avoid picking up lipids.
12. Place at -20°C for 20 min to overnight to precipitate
13. Centrifuge at high speed for 3 min
14. Decant fluid from DNA pellet and draw off remainder by pipetting (watch that the pellet does not float free from the bottom of the tube).
15. Add 200ul of 70% Ethanol and wash the pellet by gently inverting the closed tube several times, even if you have to flick the bottom of the tube to get the pellet to float free.
16. Centrifuge at medium to high speed for 2 minutes
17. Pour off ethanol and remove excess with pipette (do not dump pellet into waste)
18. Dry in oven at 65-70°C for ~5 min to remove excess Ethanol
19. Add 75ul of sterile water, 1x TE, or a low TE buffer such as Qiagen's EB buffer.
20. Incubate at 65-70°C for 1 h to overnight to resuspend DNA.

<u>Reagents/consumables</u>

DWK Life Sciences Kimble™ Kontes™ Pellet Pestle™
Use to resuspend protein and DNA pellets or grind soft tissue in microcentrifuge tubes
Supplier: DWK Life Sciences 7495200000

<u>CTAB Isolation buffer 2X</u>

Based on Coffroth et al. 1992. Marine Biology 114: 317-325

End concentrations: For 100 ml buffer mix:

1.4 M	NaCl	8.182g
20 mM	EDTA (pH 8)	0.74g
100 mM	Tris/HCl (pH 8)	1.21g
2% (w/v)	Hexadecyltrimethylammonium bromide CTAB powder	2 g
<u>Add after filter sterilization under a hood:</u>		
0.2% (v/v)	β-mercaptoethanol (<u>TOXIC!!!</u>)	200 µl

- Add ddH_2O to just under 100 ml.
- Warm to 65°C under stirring to bring the CTAB into solution.
- Once dissolved, bring final volume to 100 ml using a graduated cylinder.
- Filter sterilize (0.2 µm) into sterile 50 ml Falcon tubes and store at −20°C or 4C.

Chloroform-Ethanol DNA Purification Protocol

Reagents:

Extraction buffer (described by volume for 1 sample)

Distilled water 0.85 mL

0.5 M EDTA 0.1 mL

10% SDS (Sodium dodecyl sulfate, vendor VWR) 0.05 mL

Potassium acetate 3M pH 4.2 (adjust pH with Acetic Acid glacial)

Chloroform

Isopropanol 100%

Ethanol 70%

Procedure

1. Add 1 mL of extraction buffer to each sample, homogenize and mix well

2. Put in 72° C water bath for 12 minutes, vortex and put in another 12 minutes

   Add 2 ul of RNase and incubate 5 minutes.

   Spin for 1 min (max speed)

3. Transfer supernatant to a new microcentrifuge tube with 50 µl of potassium acetate and mix well

4. Incubate on ice for 12 minutes, vortex and incubate another 12 minutes on ice

5. Spin 12 minutes at top speed (>13000 rpm)

6. Transfer supernatant into microcentrifuge tube with 500 µl of chloroform

7. Vortex well

8. Spin 3 minutes at top speed

9. Transfer 750 µl of the top phase into a new eppendorf with 500 µl of 100% IsoOH (mixing by inverting six times)

10. Spin 6 minutes at top speed

11. Discard supernatant

12. Add 1 mL of 70% EtOH

13. Spin 6 minutes at top speed

14. Discard supernatant

15. Tap dry on paper towel

16. Dry in Speed Vac for 12 minutes

17. Dissolve DNA in 100 µl TE buffer

Preparing fly food (Opuntia Banana recipe for 1 Liter)

1L	deionized water
10g	agar
27.5g	yeast flakes
2g	Tegosept (same as Benzoic acid, p-hydroxy-, methyl ester; methyl paraben; nipagen; Lexgard M)
47.5g	Corn Syrup (we use HEB's Hill Country Light Syrup)
30g	Malt
137.5g	bananas (not too ripe; we usually peel them and freeze them)
2.125g	Opuntia powder (We are currently using "Starwest Botanicals Nopal Cactus Powder Wildcrafted, 1 Pound". We used to purchase fresh "nopalito" in cubes at the grocery store, dehydrate them in an incubator @ ~65°C, then grind them in a coffee grinder).
1.5g	Propionic Acid (add ~1ml of ethanol to dissolve; you can do it in a petri dish). The propionic acid is in a small tub under the sink. You can use the ethanol from the squirt bottle (usually in fly room).The Propionic acid plus ethanol mix is added once the food is cooling down (see below).

If (glass) vials have not been autoclaved, set the autoclave cycle **before** you begin preparing the food so that they will be ready.

Boil water for ~10 min in covered pot (you can re-use the aluminum foil to cover it)
Weigh all ingredients on Petri dishes except for corn syrup (use small beaker)

Place bananas, opuntia powder and malt in blender. With ladle take a few spoonfuls of the boiled water from the pot and add to blender. Blend and set aside.

Add agar, yeast flakes, corn syrup and Tegosept to pot with boiling water. Boil and mix with the whisk often for ~10 min. (use this time to prepare vials, to start cleaning/washing old vials, and/or to wash utensils)

Carefully pour the pot contents into the blender and blend at low speed (if you do high speed it will spill). Pour blender contents back into pot and boil (at low/med heat) for another ~10 min, mixing often.

Remove the pot from the heat and insert the food thermometer. In the meantime, add water the to the big pot and heat it up. It will be used as a water bath to keep the contents of the small pot warm while you pour vials.

Arrange the vials on clear racks or directly on the bench for pouring. They need to be visible so that you will put the correct amount.

Once the food has reached 145°F (63°C), mix in the Propionic Acid (plus ethanol).

Dispense the food into vials. We currently use a confectionery funnel ("Artilife Confectionery Funnel Stainless Steel with stand and three nozzles" to quickly dispense relatively similar amounts per vial efficiently. Othewise, you can use a ladle to serve food into a small beaker and from the small beaker pour into each vial. Refill the beaker as necessary.

Once all the food from the pot has been poured into vials, use the hot water from the large pot to begin cleaning utensils. Do not leave dirty dishes or benches.

Place the vials in the white plastic rack and cover with clean cheesecloth to prevent contamination by flies and minimize condensation. As soon as the food is settled, vials can be used. Once the vials are cool and their walls as are relatively free of condensation, cover with clean flugs, place the whole tray in a plastic bag (we reuse a white trash bag) and store in a refrigerator.

Supporting Protocol S4

Details about the pipeline followed to identify, and de novo assemble, potentially missing regions in the *s*Ald-Tx, *s*Hy2, and *s*Moj assemblies

Scheme of assemblies and reads sets used for for LASTZ alignments of our *s*Ald-Tx, *s*Hy2, and *s*Moj assemblies, and for short read mapping. Each cell indicates a separate alignment/mapping procedure.

	**Assembly used as reference ("Target sequence") for LASTZ alignment or short read mapping**					
**Procedure**	***s*Ald-Tx**	***s*Hy2**	***s*Moj**	***S. phoeniceum* GCF_003339775**	***S. kunkelii* GCF_001274875**	***S. melliferum* GCF_000236085.2**
**LASTZ Alignments**				*s*Ald-Tx<br>*s*Hy2<br>*s*Moj	*s*Ald-Tx<br>*s*Hy2<br>*s*Moj	*s*Ald-Tx<br>*s*Hy2<br>*s*Moj
**Short read mapping***	*s*Hy2	*s*Ald-Tx	*s*Ald-Tx	*s*Ald-Tx	*s*Ald-Tx	*s*Ald-Tx
	*s*Moj	*s*Moj	*s*Hy2	*s*Hy2	*s*Hy2	*s*Hy2
				*s*Moj	*s*Moj	*s*Moj

*, followed by mapped read extraction and de novo assembly of regions identified as missing

LASTZ v.1.04.15 plugin in Geneious Prime® 2024.0.7

Parameters

- Step length =20
- Seed pattern 12 of 19
- Allow single transition in see hit = no
- Perform chaining = no
- Perform gapped alignment = yes
- Search strand = Both
- HSP Threshold Score (upper limit) = 3000
- Gapped Threshold Score (upper limit) = 3000

Minimap2 v.2.17 implemented in https://galaxy-grace.hprc.tamu.edu/maroon
Parameters set:
Short reads without splicing (-k21 -w11 --sr -F800 -A2 -B8 -O12,32 -E2,1 -r50 -p.5 -N20 -f1000,5000 -n2 -m20 -s40 -g200 -2K50m --heap-sort=yes --secondary=no) (sr)

Samtools v.1.9
View parameters:
Exclude reads with any of the following flags set:
Read is unmapped
Alignment of the read is not primary
Read fails platform/vendor quality checks
Read is a PCR or optical duplicate

Geneious assembler: reads mapped to regions in need of assembly were extracted, had trimmed annotations (added by Minimap2) removed, and were filtered to retain only those with length of 100 bp (for *s*Moj) and 150bp (for *s*Ald-Tx and *s*Hy2).
Parameters in the Geneious assembler:
Mapper = Geneious
Sensitivity = Custom Sensitivity
Trim Before Assembly: Do not trim
Advanced:
Allow Gaps = yes; Maximum per read: 15%; Maximum gap size = 2
Word length = 14; Index word length = 12
Ignore words repeated more than 200 times; Reanalyze threshold = 8
Maximum mismatches per read = 3%; Maximum Ambiguity = 4
Merge homopolymer variants = yes
